# Phytochrome-interacting factors integrate environmental signals to regulate tomato growth and development

**DOI:** 10.1093/plphys/kiag379

**Published:** 2026-06-16

**Authors:** Srinivas Kunta, Abigail Aryee-Atta, Yardena Dahan, Zeenu Singh, Shlomi Aharon, Asher Pasha, Ido Nir, Ran N Lati, Nicholas J Provart, Yogev Burko

**Affiliations:** The Institute of Plant Sciences, Agricultural Research Organization (ARO), Volcani Center, Rishon LeZion 7505101, Israel; The Robert H. Smith Institute of Plant Sciences and Genetics in Agriculture, Hebrew University, Rehovot 7610001, Israel; The Institute of Plant Sciences, Agricultural Research Organization (ARO), Volcani Center, Rishon LeZion 7505101, Israel; The Robert H. Smith Institute of Plant Sciences and Genetics in Agriculture, Hebrew University, Rehovot 7610001, Israel; The Institute of Plant Sciences, Agricultural Research Organization (ARO), Volcani Center, Rishon LeZion 7505101, Israel; The Institute of Plant Sciences, Agricultural Research Organization (ARO), Volcani Center, Rishon LeZion 7505101, Israel; The Robert H. Smith Institute of Plant Sciences and Genetics in Agriculture, Hebrew University, Rehovot 7610001, Israel; Department of Plant Pathology and Weed Research, Agricultural Research Organization (ARO), Volcani Institute, Newe Ya'ar Research Center, RamatYishay 30095, Israel; Department of Cell and Systems Biology/Centre for the Analysis of Genome Evolution and Function, University of Toronto, Toronto, ON M5S 3B2, Canada; The Institute of Plant Sciences, Agricultural Research Organization (ARO), Volcani Center, Rishon LeZion 7505101, Israel; Department of Plant Pathology and Weed Research, Agricultural Research Organization (ARO), Volcani Institute, Newe Ya'ar Research Center, RamatYishay 30095, Israel; Department of Cell and Systems Biology/Centre for the Analysis of Genome Evolution and Function, University of Toronto, Toronto, ON M5S 3B2, Canada; The Institute of Plant Sciences, Agricultural Research Organization (ARO), Volcani Center, Rishon LeZion 7505101, Israel

## Abstract

Plants respond to environmental cues, such as light and temperature, which regulate their growth and development. In the model plant Arabidopsis (*Arabidopsis thaliana*), PHYTOCHROME-INTERACTING FACTORS (PIFs) are central regulators of both shade avoidance and thermomorphogenesis. However, their functional roles in crop species are less well known. Here, we generated a tomato (*Solanum lycopersicum*) mutant lacking all 8 known *PIF* genes (*slpifo*), to investigate their roles under controlled and field conditions. We showed that SlPIFs are essential for shade responses, while thermomorphogenesis-induced elongation is largely independent of SlPIFs, revealing species-specific differences in regulatory mechanisms. Under low-red to far-red light (low R/FR), *slpifo* plants failed to exhibit characteristic wild-type responses, including shoot elongation, expansion and thinning of leaf blades, and depletion of leaf chlorophyll. We further identified redundant roles for SlPIF1a, SlPIF4, and SlPIF8a in regulating stem elongation and chlorophyll depletion in response to low R/FR. In addition, *slpifo* plants exhibited reduced overall growth, fruit size, fruit number, and seed dormancy under field conditions, highlighting broader roles for SlPIFs beyond neighbor detection. These findings provide insights into how PIFs orchestrate organ-specific developmental plasticity in tomato, offering avenues to optimize light responsiveness for crop improvement.

## Teaser

Light-responsive transcription factors mediate tomato responses to shade, shaping plant architecture and modulating fruit production and seed dormancy.

## Introduction

Plants adapt to changing environmental conditions by continuously monitoring their surroundings and regulating their growth and development. This adaptability enables efficient resource acquisition and energy utilization according to prevailing growth conditions. A key factor in this process is the plant's ability to detect and respond to light, which is vital for photosynthesis and signaling functions. One such signal enables plants to detect and respond to light competition from neighboring plants through the shade-avoidance syndrome (SAS), a suite of morphological and physiological adaptations characterized by enhanced stem elongation, leaf hyponasty, reduced leaf thickness and pigment content, and accelerated flowering (Morgan and Smith 1976; Ballaré et al. 1997; Smith and Whitelam 1997; Neff et al. 2000; Casal 2012; Roig-Villanova and Martinez-Garcia 2016; Courbier et al. 2021; Casal and Fankhauser 2023; Legris 2023). These responses are mediated by the photoreceptor phytochrome B (phyB), which can sense changes in the red to far-red (R/FR) light ratio, which signals the presence of neighboring plants (Ballaré et al. 1987, 1990). The central regulators downstream of phyB are the PHYTOCHROME-INTERACTING FACTORS (PIFs), a family of basic helix–loop–helix transcription factors that orchestrate gene expression in response to light signals (Park et al. 2004; Al-Sady et al. 2006; Franklin 2008; Lorrain et al. 2008; Shen et al. 2008; Franklin and Quail 2010; Leivar and Quail 2011; Li et al. 2012; Casal 2013; Legris et al. 2019).

In addition to their role in regulating the light response, PIFs also play an important role in thermomorphogenesis, a developmental program characterized by increased stem elongation, reduced leaf thickness, and enhanced stomatal development under elevated ambient temperatures (Johansson and Hughes 2014; Jung et al. 2016; Legris et al. 2016; Sellaro et al. 2019; Legris 2023; Das et al. 2025). In the model plant *Arabidopsis thaliana*, PIF4, PIF7, and, to some extent, PIF5 regulate SAS and thermomorphogenesis (Lorrain et al. 2008; Koini et al. 2009; Franklin et al. 2011; Leivar et al. 2012; Li et al. 2012; Raschke et al. 2015; Chung et al. 2020; Fiorucci et al. 2020; Kim et al. 2020; Oh et al. 2020; Bianchimano et al. 2023; Quint et al. 2023). Their activity is modulated by phyB, whose inactivation under low R/FR or warm temperatures releases PIFs, which promote the expression of hormone and growth-related target genes, particularly those associated with cell-wall remodeling, as well as auxin biosynthesis, transport, and signaling (Oh et al. 2007; Franklin 2008; Lorrain et al. 2008; Franklin and Quail 2010; Hornitschek et al. 2012; Li et al. 2012; Casal 2013; Leivar and Monte 2014; Filo et al. 2015; Mizuno et al. 2015; Jung et al. 2016; Legris et al. 2016, 2019; Pedmale et al. 2016; Sellaro et al. 2019; Krahmer and Fankhauser 2024).

Tomato (*Solanum lycopersicum*), a model system for fleshy fruit and an important agricultural crop, exhibits adaptive responses to light and temperature similar to Arabidopsis, but with distinct regulatory nuances. For example, while auxin is sufficient to promote hypocotyl elongation in Arabidopsis, in tomato, auxin alone has limited effects on elongation. Instead, both gibberellin and brassinosteroids are necessary and sufficient to promote hypocotyl and epicotyl elongation in tomato (Li et al. 2024). In addition, while Arabidopsis leaf blade growth is restricted under shady conditions, tomato leaf growth is enhanced under the same light conditions (Carabelli et al. 2007; Chitwood et al. 2015; de Wit et al. 2015; Hussain et al. 2022).

The tomato genome encodes 8 PIF homologs: *SlPIF1a*, *SlPIF1b*, *SlPIF3*, *SlPIF4*, *SlPIF7a*, *SlPIF7b*, *SlPIF8a*, and *SlPIF8b*, which have arisen from gene duplications during its evolutionary history (Rosado et al. 2016). These homologs exhibit functional diversity, regulating processes ranging from seed germination and stem elongation to fruit development and ripening. For instance, SlPIF1a and SlPIF3 have been implicated in light-regulated fruit metabolism, while SlPIF4 is associated with cold tolerance, fruit size, and flowering time (Llorente et al. 2016; Gramegna et al. 2019; Rosado et al. 2019; Wang et al. 2020; Pan et al. 2021; Simon-Moya et al. 2021). Despite these insights, our understanding of the roles of tomato SlPIFs in regulating growth and development remains limited.

Several studies have begun to elucidate the roles of tomato PIFs under specific environmental conditions. For example, SlPIF4 has been shown to regulate hypocotyl elongation in response to both low R/FR and warm temperatures, with context-dependent variability (Rosado et al. 2019; Sun et al. 2020; Wang et al. 2020; Zhu et al. 2024). In a recent study, we demonstrated that in 21-d-old tomato plants, SlPIF8a plays a pivotal role as a regulator of the low R/FR elongation response, with redundant activity with SlPIF4, SlPIF7a, and SlPIF7b. Interestingly, under the examined growth conditions, these 4 SlPIFs were found not to be required for thermomorphogenesis-induced stem elongation in 21-d-old tomato plants grown in soil under long-day (LD), day-neutral, and short-day (SD) conditions (Kunta et al. 2025). However, other SlPIF family members may have acquired this function, especially in light of the “substitution” between the activity of Arabidopsis PIF7 and tomato SlPIF8a (Kunta et al. 2025). In this study, we aimed to characterize the roles of the SlPIF family in regulating tomato growth and development using an octuple loss-of-function mutant lacking all known SlPIF activity in combination with phenotypic analyses.

## Results

### A higher-order *SlPIF* mutant reveals functional redundancy in shade avoidance, but not thermomorphogenesis

We recently demonstrated that the shade response in 21-d-old tomato plants is predominantly regulated by SlPIF8a, with modest contributions from SlPIF4, SlPIF7a, and SlPIF7b. In contrast, at the same seedling stage, these genes were not required to promote stem elongation in response to warm ambient temperatures (Kunta et al. 2025). We speculated that the remaining SlPIFs may have acquired this role in tomato and therefore play a role in thermomorphogenesis-induced elongation. To examine this speculation, we generated a high-order mutant tomato plant that carried a loss-of-function mutation in all 8 known *SlPIF*s: *SlPIF1a*, *SlPIF1b*, *SlPIF3*, *SlPIF4*, *SlPIF7a*, *SlPIF7b*, *SlPIF8a*, and *SlPIF8b*. We named this mutant plant *slpifo* (see Materials and Methods and Fig. S1).

**Figure 1:**
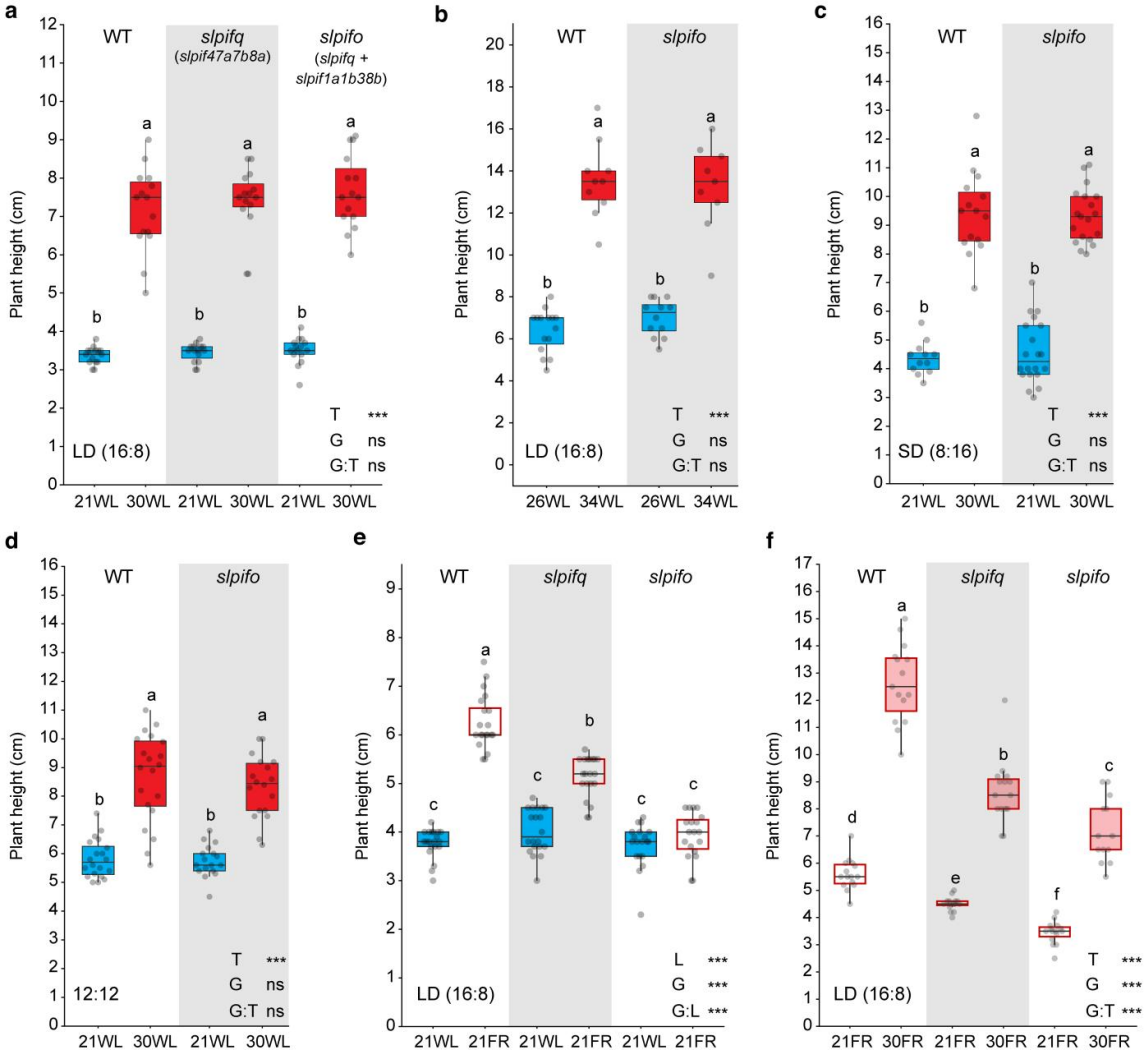
In tomato plants grown under white light, SlPIFs are not involved in shoot elongation induced by thermomorphogenesis. Heights of 21-d-old wild-type and *slpif*-mutant plants grown under a) LD conditions (16 h light/8 h dark; LD, ∼200 *µ*mol m^−2^ s^−1^), c) SD conditions (8 h light/16 h dark; SD, ∼200 *µ*mol m^−2^ s^−1^), or d) day-neutral conditions (12 h light/12 h dark; 12:12, ∼160 *µ*mol m^−2^ s^−1^) at 21 °C under white light for 9 d and then kept under the same light conditions at either 30 °C (30WL) or 21 °C (21WL). *n* > 12 plants per sample. b) Heights of 21-d-old wild-type and *slpifo* mutant plants grown under LD at 26 °C under white light (∼200 *µ*mol m^−2^ s^−1^) for 9 d and then transferred to LD at either 36 °C (36WL) or 26 °C (26WL) under white light. *n* > 9 plants per sample. e) Heights of 21-d-old wild-type and *slpif*-mutant plants grown under LD (∼200 *µ*mol m^−2^ s^−1^) at 21 °C white light for 9 d and then moved to be kept at 21 °C under white light (∼200 *µ*mol m^−2^ s^−1^) supplemented with far-red light (21FR, R/FR = 0.6) or kept at 21WL. *n* > 19 plants per sample. f) Heights of 21-d-old wild-type and *slpif*-mutant plants grown under LD (∼200 *µ*mol m^−2^ s^−1^) at 21 °C under white light for 9 d and then moved to 21 °C (21FR) or 30 °C (30FR) under white light supplemented with far-red light (R/FR = 0.6). *n* > 13 plants per sample. Boxes indicate the first and third quartiles, whiskers indicate the minimum and maximum values, black lines within the boxes indicate the median values, and gray dots indicate the individual data points. Different letters denote statistical differences (*P* < 0.05) among samples, as assessed by 2-way ANOVA and Tukey's HSD (honestly significant difference) test. T, temperature; L, light; G, genotype; G:T and G:L, interaction between genotype and temperature or light, respectively. *P < 0.05; **P < 0.01; ***P < 0.001; ns, not significant.

To evaluate the role of SlPIFs in the thermomorphogenesis-induced elongation response, we exposed 9-d-old tomato seedlings of *slpifo* mutant, along with its corresponding wild-type (*S. lycopersicum* cv. M82 *sp*), to warm ambient temperatures for an additional 12 d. Similar to what we observed for *slpifq* (mutations in *SlPIF4*, *SlPIF7a*, *SlPIF7b*, and *SlPIF8a*; described in Kunta et al. 2025), 21-d-old *slpifo* elongates like wild-type plants in response to warm temperatures (30 °C) under white light and LD (16:8), SD (8:16), or day-neutral (12:12) or under LD conditions at a higher ambient temperature (34 °C; Fig. 1a to d). However, when *slpifo* plants were exposed to low R/FR conditions at 21 °C, 24 °C, or 30 °C, they exhibited either no elongation or altered elongation, depending on the growth conditions (Figs. 1 and  S2). At 21 °C low R/FR (21FR) under LD, SD, or day-neutral conditions, they failed to elongate (Fig. 1e; compare Fig. S2b with Fig. 1c and Fig. S2c with Fig. 1d). In contrast, at 30 °C low R/FR (30FR) under LD, they exhibited altered elongation that resembled the elongation induced by warm temperature under white light (compare Fig. 1f and a). Interestingly, while *slpifq* still presents a minor response to low R/FR at 21 °C, this response was completely abolished in *slpifo*, suggesting a role for SlPIF1a, SlPIF1b, or SlPIF3. We excluded the option that SlPIF8b plays a role, as its expression is below detectable levels, and it has been shown not to participate in the response to low R/FR (Kunta et al. 2025). Therefore, to evaluate the roles of SlPIF1a, SlPIF1b, and SlPIF3 during the response to low R/FR, we generated a mutant plant with a loss-of-function mutation in all 3 genes (*slpif1a1b3*; Fig. S1). We found that the *slpif1a1b3* elongated similarly to wild-type plants in response to low R/FR (Fig. S2a to c), suggesting that these genes are not necessary for the elongation response as long as other SlPIFs remain functional. Since previous studies reported a role for SlPIF4 in the thermomorphogenic response of very young tomato seedlings grown on media right after germination (Rosado et al. 2019; Zhu et al. 2024), we tested the elongation of wild-type, *slpif1a1b3*, *slpifq*, and *slpifo* in response to warm temperature under similar conditions. We observed that while *slpif1a1b3* and *slpifq* elongated similarly to the wild-type control, *slpifo* showed moderately reduced elongation in response to warm temperature (Fig. S2d). These results contrast with our observations in 21-d-old plants grown in soil, in which *slpifo* elongated similarly to the wild type in response to warm temperature.

We conclude that under our growth conditions (21-d-old tomato plants grown in soil), SlPIFs play a key, partially redundant role in the response to low R/FR at both low and high ambient temperatures. However, they are not required for thermomorphogenesis-induced stem elongation in white light (R/FR > 1). In contrast, in 7-d-old media-grown seedlings, SlPIFs appear to function in a highly redundant manner. Nevertheless, the ability of tomato seedlings lacking all active SlPIFs (*slpifo*) to still elongate under these conditions indicates that at this stage, the thermomorphogenesis does not rely primarily on SlPIFs, similar to what we observed in 21-d-old plants. These observations imply that in tomato, additional factors contribute to the thermomorphogenesis-induced elongation response. In addition, our higher-order mutant analysis revealed that SlPIF1a, SlPIF1b, and SlPIF3 can also contribute to the elongation response to low R/FR, but only against the background of other SlPIF mutations, specifically *SlPIF4*, *SlPIF7a*, *SlPIF7b*, and *SlPIF8a*.

### SlPIFs regulate low R/FR-induced leaf morphological responses and contribute to leaf development under white light

Previous reports have shown that low R/FR causes changes in the leaf developmental program in tomato, leading to elongation of the petiole and rachis, increased blade area, and reduced leaf thickness (Chitwood et al. 2015; Courbier et al. 2021). However, the role of SlPIFs in regulating this process remained unknown. To address this, we analyzed leaf morphology in *slpif* mutant plants under low R/FR conditions. Because we focused specifically on the low R/FR response, we performed this experiment at 24 °C, the optimal temperature for tomato growth, and a condition under which *slpifo* shows strong elongation suppression (Fig. S2a). We exposed both wild-type and *slpifo* seedlings to low R/FR after the second and third leaves had already initiated from the shoot apical meristem (Fig. S3).

**Figure 2:**
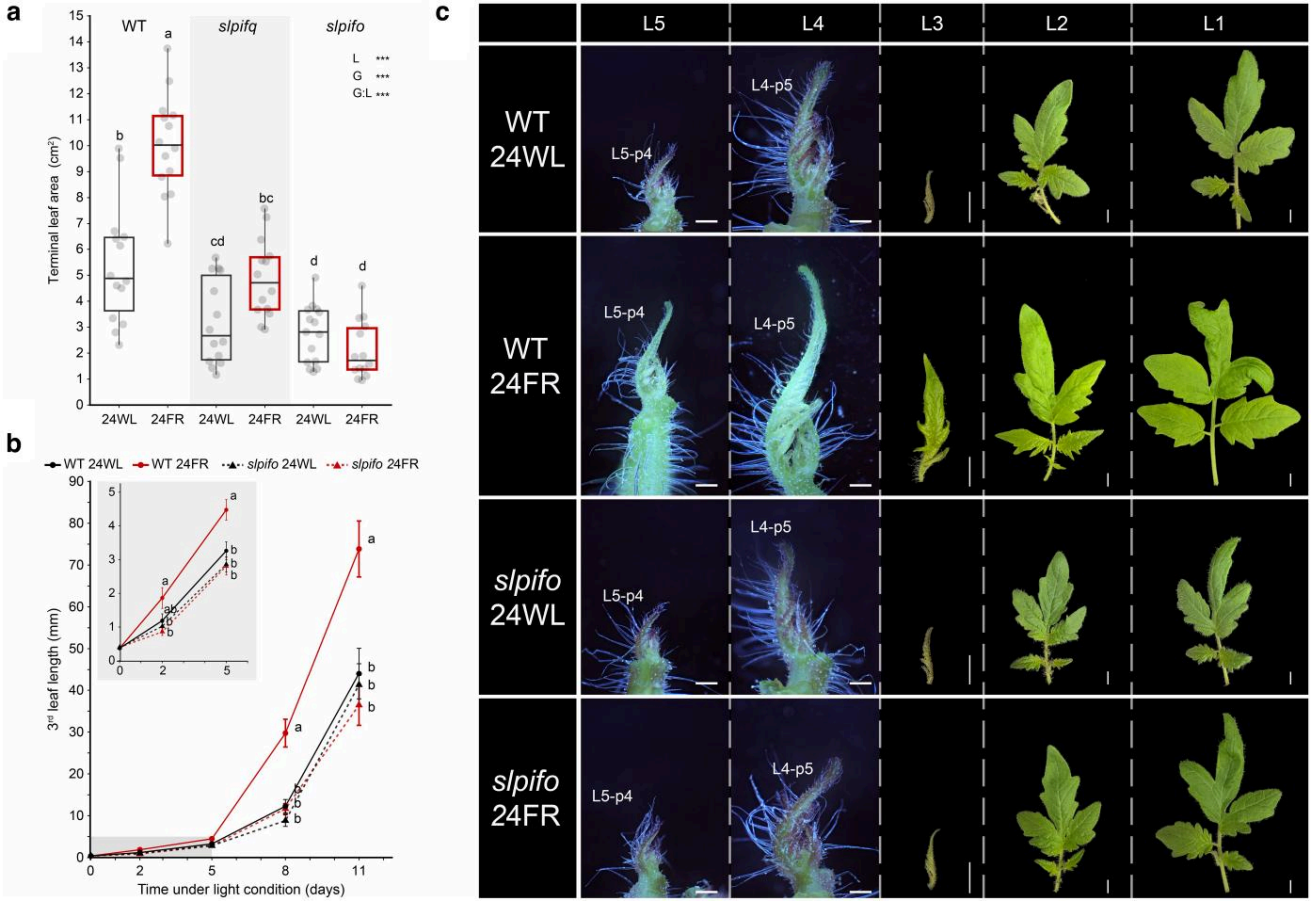
Low R/FR conditions alter leaf growth and development in an SlPIF-dependent manner. a) Area of the terminal leaflet of the third leaf from 21-d-old wild-type, *slpifq*, and *slpifo* mutant plants grown under LD (∼170 *µ*mol m^−2^ s^−1^) conditions at 24 °C under white light for 9 d (24WL) and then moved to 24 °C under white light supplemented with far-red light (24FR, R/FR = 0.6, red boxes) or kept at 24WL (black boxes). *n* > 14 plants per sample. Boxes indicate the first and third quartiles, whiskers indicate the minimum and maximum values, black lines within the boxes indicate the median values, and gray dots indicate the individual data points. Different letters denote statistical differences (*P* < 0.05) among samples, as assessed by 2-way ANOVA and Tukey's HSD. L, light; G, genotype; G:L, interaction between genotype and light. ***P < 0.001. Representative images of the terminal leaflet are shown in Fig. S4. b) Leaf growth of the third leaf of wild-type (circles, solid line) and *slpifo* mutant (triangles, dashed line) plants, represented as length at different times and under the indicated conditions. Seedlings were grown at 24WL for 9 d (Day 9 is time point 0) and then moved to 24FR (red) or kept at 24WL (black). The inset shows a magnification of the time points highlighted in gray. The average values of 7 biological replicates per condition and genotype ±Se are presented. Different letters denote statistical differences (*P* < 0.05) among samples, as assessed by 2-way ANOVA and Tukey's HSD for each time point separately. Representative images of the third leaf at the different time points are presented in Fig. S3. c) Representative images of the first true leaf (L1) through the fifth leaf (L5) at the indicated developmental stages from wild-type and *slpifo* mutant plants grown for 9 d at 24 °C under white light (24WL) and then transferred to the indicated conditions for an additional 8 d. For L4 and L5, developmental stages are indicated as plastochrons (P), which represent intervals between successive leaf primordia. P1 denotes the youngest visible leaf primordium; it becomes P2 when the next primordium is initiated, and so forth. Scale bars: 500 *µ*m for L4 and L5 and 5 mm for L1 to L3. In L1 to L3, images were digitally extracted for comparison.

**Figure 3:**
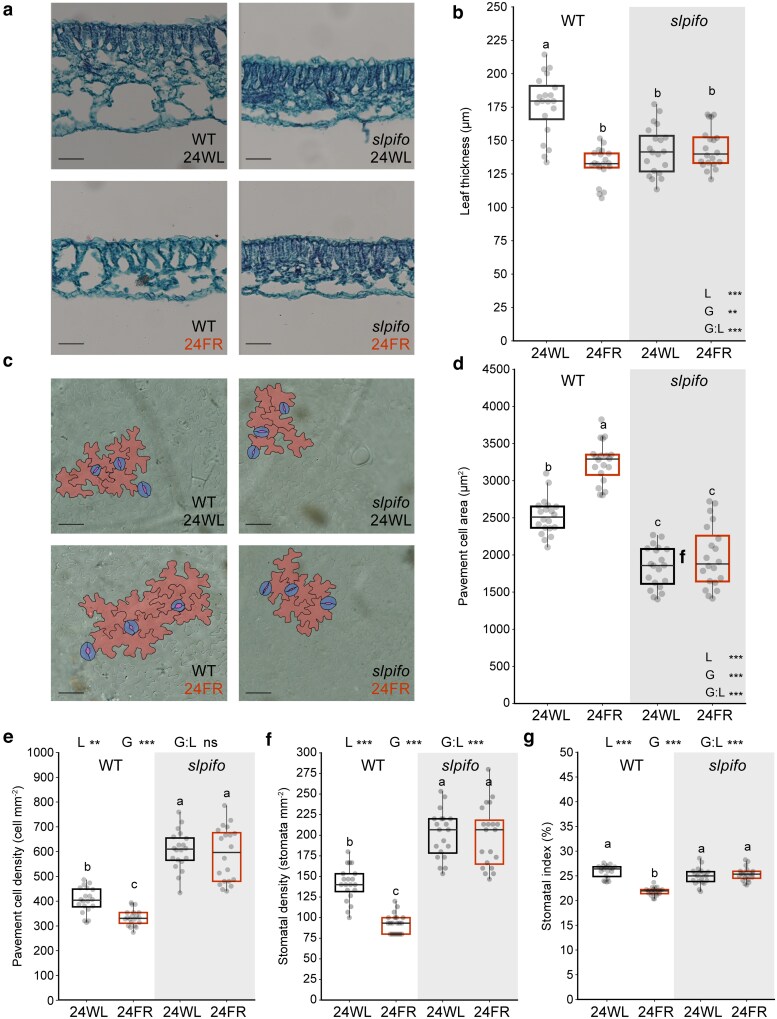
Low R/FR promotes leaf architectural changes in tomato in an SlPIF-dependent manner. a and b) Leaf thickness, c and d) pavement cell area, e) pavement cell density, f) stomatal density, and g) stomatal index. Measurements were performed on the abaxial side of the terminal leaflets of the third leaf (L3) from tomato plants exposed to either 24WL (black boxes) or 24FR (red boxes) for 12 d, as described in Fig. 2a. *n* = 20. Boxes indicate the first and third quartiles, whiskers indicate the minimum and maximum values, black lines within the boxes indicate the median values, and gray dots indicate the individual data points. Scale bars: 50 *µ*m in a) and c). In c), each image shows 7 pavement cells (coral) and 3 stomatal cells (purple), which are false-colored. Different letters denote statistical differences (*P* < 0.05) among samples, as assessed by 2-way ANOVA and Tukey's HSD. L, light; G, genotype; G:L, interaction between genotype and light. **P < 0.01; ***P < 0.001; ns, not significant. Gray dots indicate the individual data points.

Similar to previous reports (Chitwood et al. 2015; Courbier et al. 2021), we observed that in wild-type plants both the area of the terminal leaflet blade and the overall leaf length increased under low R/FR conditions (Figs. 2a to c and  S3 and S4). Remarkably, the effect of low R/FR on leaf size and shape was detectable 2 d after exposure and persisted throughout leaf development, beginning at the primordial stage (Figs. 2b and c and  S3). In addition, the thickness, as well as the dry and fresh weights of the terminal leaflet in wild-type plants decreased (Figs. 3a and b and  S5a and b). Analysis of pavement cell density in the terminal leaflet revealed an increase in cell size and a decrease in cell number in response to low R/FR, suggesting that the increase in blade area is likely a result of epidermal cell expansion (Fig. 3c to e). Interestingly, stomatal density and the stomatal index decreased in response to low R/FR (Fig. 3f and g). We observed that the increase in terminal leaflet area in response to low R/FR was completely abolished in *slpifo* (*slpifq* + *slpif1a1b38b*) and only partially reduced in *slpifq* (Figs. 2a and  S4). Therefore, to evaluate the role of SlPIFs in leaf development, we focused primarily on *slpifo*.

Strikingly, in the *slpifo* mutant, all of the leaf phenotypes observed in wild-type plants in response to low R/FR, including increased terminal leaflet area and leaf length, reduced thickness and biomass, larger pavement cells, and decreased stomatal density and index, were absent (Figs. 2 and 3 and  S3 to S5). In fact, *slpifo* leaves exhibited subtle developmental changes under white light, including a smaller leaf blade area, reduced leaf thickness, and smaller pavement cells accompanied by increased pavement cell densities and stomatal densities (Figs. 2 and 3). These observations suggest that, in addition to their central role in the response to low R/FR, SlPIFs also contribute to leaf development independently of the shade-avoidance response.

Altogether, our findings indicate that in response to low R/FR, SlPIFs regulate diverse morphological changes, including increased terminal leaflet area and leaf length, reduced leaf thickness, and decreased stomatal density. These results highlight the central role of this transcription factor family, extending beyond stem elongation, in orchestrating tomato's response to low R/FR and regulating plant development.

### Chlorophyll depletion in shade-avoiding leaves is redundantly regulated by SlPIF1a, SlPIF4, and SlPIF8a

In addition to changes in leaf growth and development, we observed that following exposure to low R/FR, the leaves developed a pale green color, unlike that of the leaves of plants grown under white light. This shade-induced phenotype has been observed in many plant species, including *Cardamine hirsuta*, *A. thaliana*, *Rumex obtusifolius*, and *S. lycopersicum* (McLaren and Smith 1978; Smith and Whitelam 1997; Cagnola et al. 2012; Patel et al. 2013; Kalaitzoglou et al. 2019; Molina-Contreras et al. 2019; Jawahir et al. 2026).

**Figure 4:**
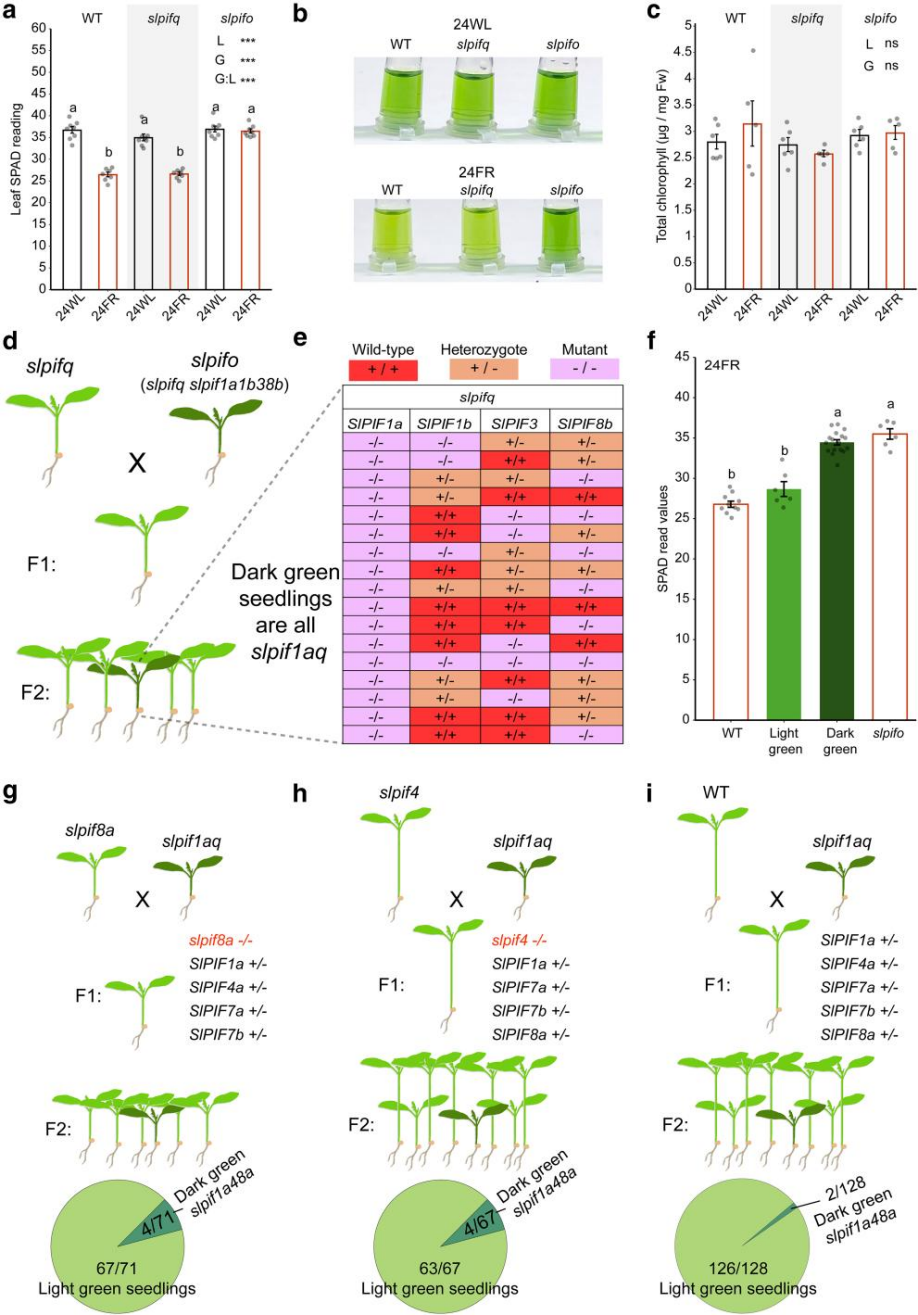
Chlorophyll depletion under low R/FR light conditions is regulated by SlPIFs in a highly redundant manner. a) Chlorophyll content per unit of leaf area measured using SPAD, b) representative images of the chlorophyll extraction, and c) total chlorophyll content per unit of fresh weight in the indicated genotypes and under the indicated conditions. Measurements were performed on the terminal leaflets of the third leaf (L3) from tomato plants exposed to either 24WL (black bars) or 24FR (red bars) for 12 d, as described in Fig. 2a. The average values ± Se are presented. *n* > 5. Different letters denote statistical differences (*P* < 0.05) among samples, as assessed by 2-way ANOVA and Tukey's HSD. L, light; G, genotype; G:L, interaction between genotype and light. ***P < 0.001; ns, not significant. Gray dots indicate the individual data points. d) Schematic illustration of the segregating population developed by crossing a homozygous *slpifq* line with a homozygous *slpifo* line and grown under low R/FR light conditions. Heterozygous plants were then self-pollinated to produce a segregating F_2_ population. e) Genotyping of the dark green (*slpifo*-like) plants identified in the F_2_ generation, shown in d), for *SlPIF1a*, *SlPIF1b*, *SlPIF3*, and *SlPIF8b*. Note that all of the dark green individuals were homozygous for the *slpif1a* mutation and segregated at all of the other tested loci. +/+ wild type; +/− heterozygous; −/− mutant. f) Chlorophyll content per leaf area (measured using SPAD) in *slpifo*, wild-type, and representative F_2_ dark green or light green individuals, as shown in d). The average values ± Se are presented. *n* > 6. Different letters denote statistical differences (*P* < 0.05) among samples, as assessed by 1-way ANOVA and Tukey's HSD. Gray dots indicate the individual data points. g to i) Schematic illustrations of the segregating population developed by crossing *slpif1aq* (*slpif1a47a7b8a*), an individual isolated from the F_2_ population described in d), with the indicated genotypes. Heterozygous F_1_ plants were then self-pollinated to produce segregating F_2_ populations. The segregating genes in each population are indicated next to the corresponding F_1_ plant. The pie chart below each population indicates the number of dark green (*slpifo*-like) and light green plants identified out of the total F_2_ plants grown under 24FR conditions.

In Arabidopsis, chlorophyll reduction in response to shade conditions is dependent on PIF1, PIF3, PIF4, and PIF5. In addition, PIF1 and PIF3 have been shown to regulate chlorophyll biosynthesis negatively during de-etiolation (Huq et al. 2004; Moon et al. 2008; Stephenson et al. 2009; Toledo-Ortiz et al. 2010, 2014; Morelli et al. 2021). In contrast, in rice (*Oryza sativa*), OsPIL1 (considered a homolog of PIF4 and PIF5) may promote chlorophyll biosynthesis (Nakamura et al. 2007; Sakuraba et al. 2017). These previous findings led us to investigate the role of SlPIF in the chlorophyll-depletion phenotype during tomato's response to low R/FR. Using chlorophyll extraction and soil-plant analysis development (SPAD) measurements, we observed a significant reduction in chlorophyll levels per unit of leaf area following exposure to low R/FR light in wild-type plants (Figs. 4a and b and  S5c). In contrast, *slpifo* mutants maintained similar chlorophyll levels under both white-light and low R/FR conditions (Figs. 4a and b and  S5c). After normalizing chlorophyll levels to fresh tissue weight to account for the differences observed in leaf thickness and cell size, no significant differences were detected between the response to white light and low R/FR or among genotypes (Fig. 4c), consistent with a previous study that examined wild-type tomato leaf response to low R/FR (Cagnola et al. 2012).

These results suggest that the observed reduction in chlorophyll levels per unit of leaf area may be caused by a dilution effect resulting from leaf expansion (Figs. 2a and 3c and d), a phenomenon that has been described previously in leaves exposed to supplemental far-red light (McLaren and Smith 1978; Li and Kubota 2009; Mickens et al. 2018; Zhen and Bugbee 2020; Kelly and Runkle 2024). We cannot exclude the possibility that the depletion in chlorophyll levels in the leaf is the result of a direct effect on chlorophyll biosynthesis. However, given the observed changes in leaf morphology and the lack of differential expression of genes involved in chlorophyll biosynthesis (*CHLM, POR1,* and *HEMA2*) and light-harvesting antenna complexes (*CAB13* and *LHCB3*) normally associated with chlorophyll accumulation, this possibility appears less likely (Fig. S6a; see also transcriptomic analysis: https://bar.utoronto.ca/efp_tomato/cgi-bin/efpWeb.cgi?dataSource=Shade_Timecourse_WT; Kunta et al. 2025).

Surprisingly, in contrast to the stem elongation and leaf development responses to low R/FR, for which the *slpifq* phenotype was detectable, but less robust than it was in *slpifo*, the reduction in chlorophyll per unit of leaf area in *slpifq* mutants was indistinguishable from that observed in wild-type plants (Figs. 4a and b and  S5c). These observations suggest a high degree of functional redundancy among SlPIFs in regulating chlorophyll depletion, unlike low R/FR-induced stem elongation, for which the *slpif8a* mutant alone was sufficient to suppress most of the response (Kunta et al. 2025).

To further explore the redundant role of SlPIFs in regulating leaf chlorophyll levels and to identify the minimal set of SlPIF members required to control the chlorophyll-depletion phenotype, we crossed *slpifo* plants with *slpifq* plants and screened the F_2_ segregating population for individuals that remained dark green under low R/FR, as observed for the *slpifo* mutant (Fig. 4d). We found that 17 out of 72 plants exhibited an *slpifo*-like phenotype (small and dark green), suggesting single-gene segregation. Genotyping of all *slpifo*-like plants revealed that they all carried a homozygous loss-of-function mutation in *SlPIF1a* and segregated for *SlPIF1b*, *SlPIF3*, and *SlPIF8b* (Fig. 4e). These findings suggest that when *SlPIF4*, *SlPIF7a*, *SlPIF7b*, and *SlPIF8a* are inactive (as in the *slpifq* mutant), *SlPIF1a* plays a key role in negatively regulating chlorophyll levels per unit leaf area under low R/FR.

Next, we asked whether all of the *SlPIFs* in *slpifq* (*SlPIF4*, *SlPIF7a*, *SlPIF7b*, or *SlPIF8a*) are necessary for the chlorophyll-depletion phenotype. To answer that question, we crossed *slpif1aq* with wild-type, *slpif4*, or *slpif8a* plants and again screened the F_2_ segregate population for plants with an *slpifo*-like phenotype (Fig. 4g to i). Analysis of all 3 populations suggested a 3-gene regulation. Among the offspring of the cross in which *slpif8a* was fixed (*slpif1aq* × *slpif8a*), 4 out of 71 plants had an *slpifo*-like phenotype (2-gene segregation; Fig. 4g). Among the offspring of the cross in which *slpif4* was fixed (*slpif1aq* × *slpif4*), 4 out of 67 plants had an *slpifo*-like phenotype (2-gene segregation; Fig. 4h), and among the *slpif1aq* × wild-type (WT) offspring, 2 out of 128 plants had an *slpifo*-like phenotype (3-gene segregation, Fig. 4i). Genotyping all of the *slpifo*-like plants from the 3 populations revealed that they all carried the triple mutation *slpif1a48a*, while segregating for all other genes, depending on the nature of the cross.

To confirm that *slpif1a48a* and *slpif1aq* indeed mimic the *slpifo* phenotype, we collected seeds from homozygous plants and evaluated their response to low R/FR. As observed for *slpifo*, both *slpif1a48a* and *slpif1aq* maintained similar chlorophyll levels per leaf area under both white-light and low R/FR conditions (Fig. S6b). After normalizing chlorophyll levels to fresh tissue weight, no significant differences were detected between white-light and low R/FR treatments in *slpif1a48a* and *slpif1aq*, similar to wild type and *slpifo* (Fig. S6c). Moreover, similar to *slpifo*, both *slpif1a48a* and *slpif1aq* failed to elongate in response to low R/FR and showed a stronger impairment in elongation than *slpifq* (Fig. S6d). Finally, to confirm that *slpif1a48a* and *slpif1aq* mimic *slpifo* at the level of gene expression, we examined the expression of *ARABIDOPSIS THALIANA HOMEOBOX2* (*ATHB2*) genes, which are activated by SlPIFs in response to low R/FR (Kunta et al. 2025). As expected, the activation observed in the wild type was completely abolished in *slpif1a48a*, *slpif1aq*, and *slpifo* (Fig. S6a), whereas it was only partially repressed in *slpifq* (Kunta et al. 2025)

Together, these results suggest that the low R/FR response in tomato, including chlorophyll depletion and stem elongation, is tightly and redundantly controlled by SlPIF1a, SlPIF4, and SlPIF8a. Given the increase in leaf blade area (controlled by SlPIFs) and the lack of change in normalized chlorophyll levels during the response to low R/FR, we suggest that SlPIFs likely regulate chlorophyll levels in the leaf through regulation of the leaf morphology, which leads to dilution of chlorophyll per unit of leaf area.

### SlPIFs regulate plant architecture, fruit development, and seed dormancy in natural light environments

While our experiments thus far have focused on relatively young plants (3 to 4 wk old), previous studies have shown that SlPIF1, SlPIF3, and SlPIF4 also function in mature tomato plants (Llorente et al. 2016; Gramegna et al. 2019; Rosado et al. 2019; Pan et al. 2021; Simon-Moya et al. 2021). In addition, a shade-tolerant tomato introgression line has shown improved fruit quality and higher yields (Burbano-Erazo et al. 2025). Therefore, to evaluate the phenotypes of mature tomato plants lacking all known SlPIFs under natural growth conditions, we grew *slpifo* mutant plants alongside wild-type plants under commercial-like conditions in both greenhouse and open-field environments, including seed sowing in a commercial nursery prior to transplanting (see Materials and Methods).

**Figure 5:**
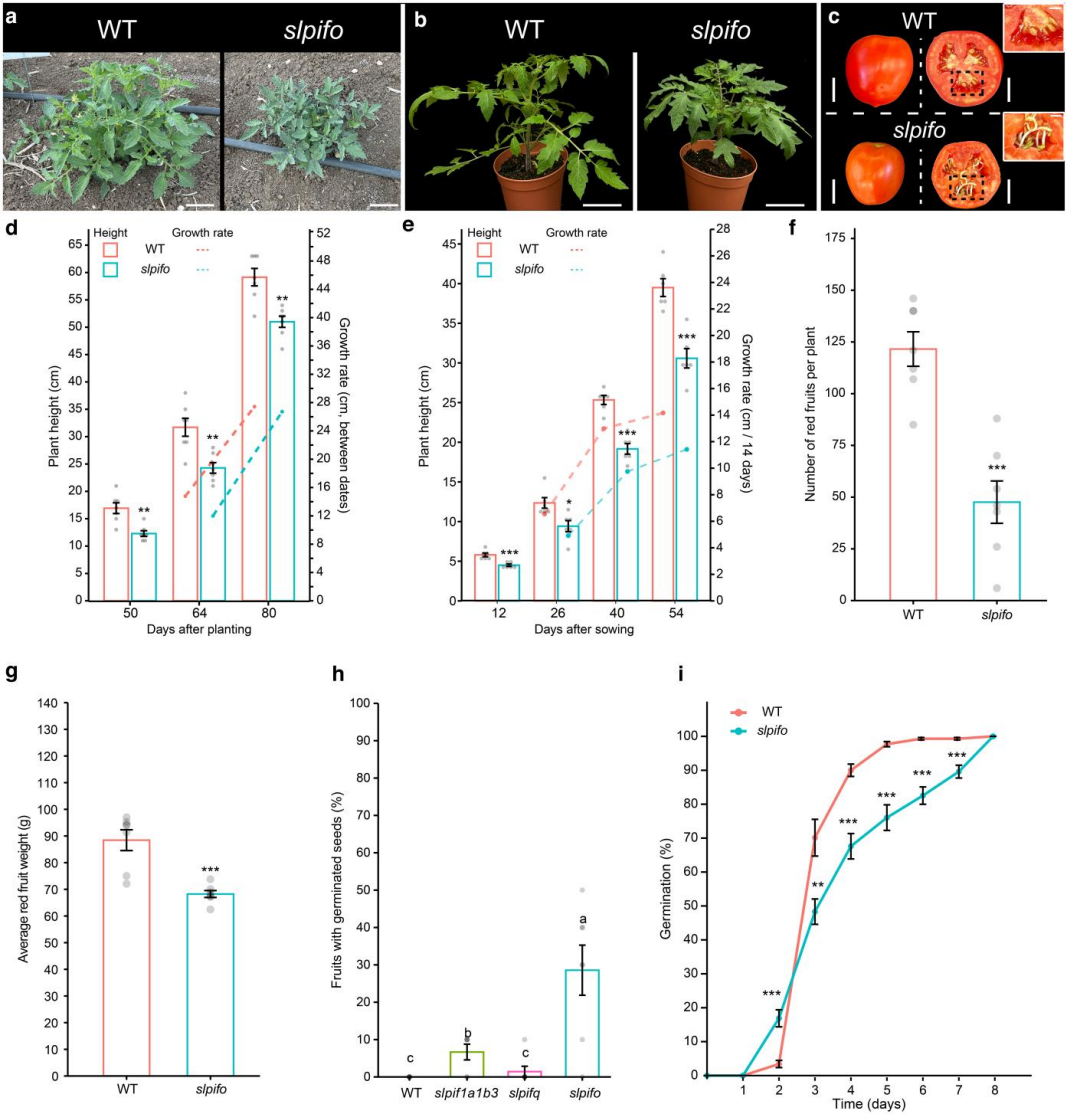
Tomato plants lacking all active *SlPIFs* exhibited slow growth, reduced fruit size and number, and increased seed germination inside the fruit. Representative phenotypes of mature wild-type and *slpifo* plants and fruits grown under a and c) field conditions or b) in the greenhouse. The inset in c) shows a magnification of the area marked by the black dashed line. In b) and c), images were digitally extracted for comparison. Scale bars: 10 cm in a) and b), 2 cm in c), and 0.5 cm in the insets. Plant heights (left *y*-axis) and growth rates (right *y*-axis) of wild-type and *slpifo* plants grown under d) open-field or e) greenhouse conditions. The average values of at least 6 plants per genotype ±Se are shown. Gray dots indicate the individual data points. f) Number of red fruits per plant, g) average red fruit weight, h) percentage of fruits containing germinated seeds inside the fruits (vivipary), from wild-type and the indicated genotypes grown in the field. The average values of at least 7 biological replicates per genotype ±Se are presented. i) kinetics of seed germination. The average values of 12 biological replicates per genotype ±Se are presented, derived from 6 independent seed batches for each genotype. Only seeds that germinated by the end of the experiment were included in the analysis (see Fig. S8d for the percentage of germinated seeds relative to the total number of seeds used). In d) to g) and i), *P < 0.05; **P < 0.01; ***P < 0.001, according to Student's *t*-test. In h), different letters denote statistical differences (*P* < 0.05) among samples, as assessed by 1-way ANOVA and Tukey's HSD. In d) to h), gray dots indicate the individual data points.

In contrast to controlled white-light environments, in which the heights of *slpifo* plants were no different from those of wild-type plants (Fig. 1), *slpifo* plants were shorter under both greenhouse and open-field conditions (Fig. 5a, b, d, and e). Growth-rate analysis over time revealed that *slpifo* plants grew more slowly in both environments (Fig. 5d and e). These observations suggest that the reduced plant height under natural conditions likely results from a direct role of SlPIFs in promoting growth in natural light, consistent with their contribution to leaf development phenotypes observed under controlled white-light conditions (Figs. 2 and 3). In the open field, the shorter phenotype of *slpifo* may also be influenced by far-red light reflected from neighboring plants (Fig. S7a), which may contribute to elongation at later developmental stages in the wild type. In contrast, in the greenhouse, the spacing between plants was sufficient to ensure that the R:FR ratio did not drop below 1 (Fig. S7b), suggesting that under these conditions, there was no substantial effect of far-red reflected from neighboring plants on the phenotype we observed.

Next, we evaluated the effect of *SlPIFs* on fruit and seed setting and found that the *slpifo* mutant produced fewer and smaller fruits (Figs. 5c, f, and g and  S8a and b). We did not detect any effect of *slpifo* on the seed weight per fruit (Fig. S8c). However, while extracting the seeds from the fruits, we noticed that in about 30% of the fruits, the seeds began to germinate within the fruits while still attached to the maternal tissue, a condition called vivipary (Eyster 1931; Fig. 5c and h). Since in Arabidopsis, PIF1, and at higher temperatures, PIF3 and PIF5 have been shown to act as repressors of seed germination (Oh et al. 2007; Piskurewicz et al. 2023), we also evaluated vivipary in *slpif1a1b3* and *slpifq* mutant plants. We found that about 10% of *slpif1a1b3* fruits contained germinated seeds, compared to 30% of the *slpifo* fruits. We rarely observed this phenomenon in *slpifq* mutant fruits (Fig. 5h). Therefore, we speculate that in addition to SlPIF1a, SlPIF1b, and SlPIF3, other SlPIFs also contribute to repressing seed germination inside the fruit. Interestingly, we observed this phenotype mainly among fruits from field-grown plants and not among fruits from greenhouse-grown plants, suggesting that this phenomenon is highly dependent on growth conditions.

To evaluate the direct effect of SlPIF on seed dormancy after extraction, we conducted a germination assay on agar medium. Wild-type seeds germinated in a well-synchronized manner, with most seeds germinating from Days 2 to 5. In contrast, *slpifo* seeds germinated asynchronously over a period of 8 d (Fig. 5i). Notably, a small subset of *slpifo* seeds germinated earlier than the wild type, whereas the majority germinated later. Because the germination kinetics analysis included only seeds that germinated by the end of the experiment, we also assessed the proportion of seeds that germinated out of the total number of seeds plated. This analysis revealed that a relatively high proportion of *slpifo* seeds failed to germinate (Fig. S8d). We speculate that these nongerminated seeds may have initiated germination within the fruit but arrested their development just before radicle emergence from the seed coat and therefore appear morphologically similar to normal seeds. To test this, we carefully examined *slpifo* seeds under a stereomicroscope and identified 2 groups: seeds with a closed cap and seeds with an open cap. As we speculated, the embryos in the open-cap seeds had already initiated germination (Fig. S9a to i). After separating the open-cap and closed-cap seeds and germinating them separately, we found that the majority of open-cap seeds failed to germinate (only 8.5% germinated), whereas most closed-cap seeds (82%) germinated successfully (Fig. S9j). These findings suggest that *SlPIF* regulates seed dormancy at both the fruit and dry seed stages, thereby affecting overall seed germination.

Altogether, these results suggest that plants lacking all active SlPIFs fail to grow normally under greenhouse or open-field conditions, indicating that their role extends beyond shade-sensing and neighbor detection. SlPIFs affect elongated growth under natural light, fruit setting, fruit development, and the repression of seed germination to ensure germination under the right conditions and at the right time.

## Discussion

PIFs are key transcriptional regulators that integrate light and other environmental signals to modulate plant growth and development across diverse species. We previously showed that SlPIF8a plays a key role in the low R/FR response in tomato and proposed that the *SlPIF* gene family may not be required for tomato thermomorphogenic elongation (Kunta et al. 2025). Here, we extend that work and demonstrate that tomato plants lacking all known SlPIFs (*slpifo*) exhibit wild-type-like elongation responses to warm temperatures under white light in 21-d-old soil-grown plants (Fig. 1). Given that tomato evolved in regions with generally warmer climates than those in which Arabidopsis evolved, tomato may have developed an alternative regulatory mechanism for thermomorphogenesis-induced stem elongation in which SlPIFs do not play a significant role.

Previous studies reported that SlPIF4 plays a role in the response to warm temperatures during the early seedling stage (7 to 8 d old) (Rosado et al. 2019; Zhu et al. 2024). Although *slpifq* seedlings, which include the *slpif4* mutation, did not show reduced elongation in response to warm temperatures in our growth conditions and at a similar developmental stage (7-d-old seedlings), *slpifo* seedlings did exhibit reduced elongation under these conditions, suggesting that SlPIFs can contribute to thermomorphogenesis at early developmental stages. However, unlike in Arabidopsis, where the elongation response is fully dependent on PIF4 and PIF7 (Koini et al. 2009; Fiorucci et al. 2020), tomato *slpifo* seedlings still exhibit a significant elongation response to warm temperatures (Fig. S2d). Notably, the effect observed in young seedlings may also partially result from altered seed dormancy or germination dynamics in the *slpifo* mutant (Figs. 5 and  S9), which could influence early seedling growth.

Overall, our observation that SlPIFs are not required for thermomorphogenic elongation in mature 21-d-old tomato plants highlights species-specific differences in the regulatory networks controlling elongation in response to warm temperatures. In Arabidopsis, additional growth-regulatory factors, including EARLY FLOWERING 3, ELONGATED HYPOCOTYL 5, CONSTITUTIVELY PHOTOMORPHOGENIC 1, and heat stress transcription factors, have been shown to play central roles in thermomorphogenesis elongation responses alongside PIFs (Delker et al. 2014; Park et al. 2017; Jung et al. 2020; Bakery et al. 2024; Xu et al. 2025). It is therefore plausible that these factors may contribute more prominently than SlPIFs to thermomorphogenic growth in tomato. Further investigation will be required to determine whether these factors or other signaling modules act as key integrators of temperature responses in this species. In addition, while we mainly focused on the role of SlPIFs during the thermomorphogenesis-mediated elongation response, given the effects of SlPIFs and warm temperatures on leaf development, including leaf thickness and stomatal patterning, future work should address whether SlPIFs are involved in thermomorphogenesis in this context (Lau et al. 2018; Legris 2023; Nir et al. 2023; Hofmann et al. 2025).

We used the *slpifo* mutant to deepen our understanding of the role of SlPIFs in regulating growth and development beyond shoot elongation in response to low R/FR. We found that SlPIFs act redundantly to regulate a complex suite of morphological and physiological responses under low R/FR conditions and during growth under natural light. For example, we identified that SlPIF1a, SlPIF4, and SlPIF8a are all required and act redundantly to negatively regulate chlorophyll levels per unit leaf area under low R/FR (Fig. 4), in contrast to shoot elongation, for which a mutation in *SlPIF8a* alone is sufficient to suppress a significant fraction of the elongation response (Kunta et al. 2025). Interestingly, SlPIF1a and SlPIF1b have been shown to promote chlorophyll biosynthesis both during de-etiolation and in light-grown tomato plants (Simon-Moya et al. 2021). Similarly, in rice, OsPIL1 (the PIF4 homolog) promotes chlorophyll biosynthesis (Sakuraba et al. 2017). In contrast, in Pennycress (*Thlaspi arvense*), PIF7 has been shown to negatively regulate chlorophyll levels in shade and, in Arabidopsis, PIF1, PIF3, and, to some extent, PIF4 and PIF5 negatively regulate chlorophyll biosynthesis during de-etiolation (Huq et al. 2004; Moon et al. 2008; Stephenson et al. 2009; Toledo-Ortiz et al. 2010, 2014; Morelli et al. 2021; Jawahir et al. 2026).

These observations suggest that the role of PIFs in regulating chlorophyll levels is both context- and species-dependent. This may reflect the dual role of PIFs in controlling both cell size and chlorophyll biosynthesis, as chlorophyll content can be modulated directly through biosynthetic pathways or indirectly through changes in cell size. It is worth noting that the relationship between shade and chlorophyll content can also be influenced by additional factors, including light intensity and the developmental stage of the leaf (Hersch et al. 2014).

Our analyses also uncovered a complex role for SlPIFs in controlling leaf thickness. In response to low R/FR light, wild-type plants exhibit a reduction in leaf thickness, a response that is abolished in the *slpifo* mutant. This indicates that SlPIFs mediate the light-induced formation of thinner leaves. However, *slpifo* plants also display thinner leaves under white light, suggesting that SlPIFs are also required to promote leaf thickness under unshaded conditions. These findings highlight the context-dependent role of SlPIFs in regulating leaf development: Under white light, they promote thicker leaves, whereas under low R/FR, they promote the formation of thinner leaves. In addition, our findings in tomato contrast with observations in Arabidopsis, for which low R/FR has been shown to suppress leaf primordium growth and leaf blade expansion via PIFs (Carabelli et al. 2007; de Wit et al. 2015; Hussain et al. 2022). In tomato, however, SlPIFs appear to promote leaf expansion, as *slpifo* mutants exhibited reduced leaf area and epidermal cell expansion under both white light and low R/FR (Figs. 2a and 3d). Interestingly, at 16 °C, Arabidopsis leaf area has been shown to increase in response to low R/FR (Patel et al. 2013). This species-specific and presumably condition-specific divergence suggests that the PIF-regulated gene networks controlling leaf growth have undergone functional differentiation, possibly reflecting adaptations to distinct ecological niches or developmental programs.

Manipulating PIF activity has often been proposed as a strategy to enhance crop performance under dense planting conditions (Huber et al. 2021; Cordeiro et al. 2022; Lyu et al. 2023; Martinez-Garcia and Rodriguez-Concepcion 2023). However, under both greenhouse and open-field conditions, complete loss of SlPIF function leads to broad developmental abnormalities, including delayed growth, altered seed dormancy, and decreased fruit size and number (Figs. 5 and  S8 and S9). Some of these phenotypes have already been reported in *SlPIF4*-silenced plants (Rosado et al. 2019). These findings suggest that transforming a plant from shade-sensitive to shade-insensitive may require parallel genetic modifications to compensate for the detrimental effects of PIF loss on reproductive development.

Taken together, our findings position SlPIF transcription factor family as central regulators of tomato development, mediating the integration of light and potentially other environmental signals into growth and development. Understanding and manipulating PIF function may offer strategies to optimize plant architecture, improve resource-use efficiency, and enhance resilience to abiotic stresses in agricultural systems. However, these matters need to be approached with caution. Modifying plant responses to neighbor proximity through PIF dysregulation can lead to unintended developmental outcomes. Complete suppression of the shade-avoidance response, as observed in *slpifo* plants, results in semi-dwarf phenotypes accompanied by abnormal development.

Moreover, during domestication, certain components of the shade-avoidance response were selected against, as reduced elongation and more compact plant architecture are generally advantageous in agricultural systems. Nevertheless, specific elements of this response may positively contribute to yield under particular environmental or management conditions. Indeed, shade responses have been associated with beneficial effects on yield and fruit quality (Kalaitzoglou et al. 2019; Burbano-Erazo et al. 2025). Therefore, more refined approaches are needed to optimize yield, aiming to modulate rather than eliminate shade-avoidance responses. For example, temporally and spatially restricted or enhanced expression of shade-promoting regulators might allow exploitation of beneficial traits while minimizing adverse effects on overall plant growth and reproductive performance. In addition, the different *slpif* mutant combinations generated in this study and in previous work, together with wild tomato varieties that retain a more complete shade-avoidance response and may serve as sources of beneficial alleles, provide valuable genetic resources for testing such targeted strategies (Bush et al. 2015; Burbano-Erazo et al. 2025; Kunta et al. 2025). Further studies will be required to evaluate the feasibility, stability, and yield outcomes of these approaches under field conditions.

## Materials and methods

### Growth conditions, plant material, and phenotyping

*S. lycopersicum* cv. M82 (*sp*) was the background cultivar for all of the experiments. For the low R/FR and warm ambient temperature experiments on soil, tomato seeds were germinated in standard growing soil in a growth chamber equipped with LED white light, under long day (LD, 16 h light/8 h dark) conditions and white light (WL, ∼160 to 200 *µ*mol m^−2^ s^−1^; see Fig. S10) for 9 d. At this stage, only seedlings with similar germination timing were retained for further analysis and either kept under the same light conditions (WL) or transferred to an environment in which they were exposed to WL supplemented with far-red light during the daytime (FR, ∼160 to 200 *µ*mol m^−2^ s^−1^, R/FR = 0.6, Fig. S10) for different periods of time. All temperatures were kept constant throughout the day and night. For the warm ambient temperature experiment using media-grown seedlings, tomato seeds were grown on plates containing Nitsch medium (4.4 g/L; Duchefa, M0224) with 2% sucrose (w/v) and kept under LD white light (∼200 *µ*mol m^−2^ s^−1^, Fig. S10b) at 24 °C for 3 d. Germinated seedlings were then transferred to either 32 °C (32WL) or maintained at 24 °C (24WL) under constant white-light conditions (∼200 *µ*mol m^−2^ s^−1^, Fig. S10b) for an additional 4 d. Plant height and hypocotyl length were measured with a ruler at the end of each experiment.

For the open-field experiments, tomato seeds were sown in flats and grown in a commercial nursery (Hishtil, Israel). After 4 wk, the plants were transplanted directly into the field at the Newe Ya’ar Research Center in Israel during the summer growing seasons. For the greenhouse experiments, tomato seeds were sown in 10-L pots and grown under greenhouse conditions at the Volcani Institute in Rishon LeZion, Israel. The greenhouse-grown and field-grown plants were grown with drip irrigation and standard fertilizer regimes, and light spectra were recorded once or twice during the growing period (see Fig. S7). For each genotype, data on plant height, fruit size, fruit number, and germination were collected from at least 10 plants.

The Golden Gate assembly method was used (Werner et al. 2012; Brooks et al. 2014) to generate a binary vector containing a CRISPR/Cas9 genome-editing cassette with *35S_pro_:Cas9* and 6 to 8 guide RNAs (gRNAs), as described in Kunta et al. (2025). To generate the *slpifo* (mutations in all 8 *SlPIF* genes), a binary vector containing 8 gRNAs targeting *SlPIF1a*, *SlPIF1b*, *SlPIF3,* and *SlPIF8b* (2 guides against each gene, Fig. S1a) was transformed into *slpifq* (*slpif47a7b8a*) plants, described in Kunta et al. (2025). To generate the *slpif1a1b3* triple-mutant, a binary vector containing 6 gRNAs targeting *SlPIF1a*, *SlPIF1b*, and *SlPIF3* (2 guides against each gene, Fig. S1) was transformed into M82 (*sp*) plants. *Agrobacterium tumefaciens* GV3101 and the cotyledon transformation method (McCormick 1997) were used for the plant transformation. Transgenic plants were screened for mutations using PCR and Sanger-sequenced to determine mutant identity. Segregation analysis of antibiotic resistance was used to isolate single-insertion homozygous transgenic lines. Specific primers for the Cas9 sequence and the surrounding gRNA target sites were used to isolate *slpif* mutants that were free of the CRISPR/Cas9 genome-editing cassette (Table S1). All assays were conducted using T3 or T4 seedlings.

The *slpif* mutant alleles used in this study were as follows: *slpif1a^CR-1^* (in *slpifo*) and *slpif1a^CR-2^* (in *slpif1a1b3*), *slpif1b^CR-1^* (in *slpifo*) and *slpif1b^CR-2^* (in *slpif1a1b3*), *slpif3^CR-1^* (in *slpifo* and *slpif1a1b3*), and *slpif8b^CR-3^* (in *slpifo*). To generate the *slpif1aq* plants, an *slpifo* plant was crossed with an *slpifq* plant. To generate the *slpif1aq* segregating population (Fig. 4), the *slpif1aq* plants were crossed with M82, *slpif4^CR-1^*, or *slpif8a^CR-1^* plants (described in Kunta et al. 2025).

### Chlorophyll measurements

For chlorophyll measurements, chlorophyll was extracted from leaf discs (10.8 mm diameter) from the terminal leaflet of the third leaf. Fresh weights were measured, and the tissue was immediately frozen in liquid nitrogen. After homogenization, the chlorophyll was extracted twice in 1 mL of ice-cold 80% acetone, and debris was removed by centrifugation at 10,000 × *g* for 20 min at 4 °C. Chlorophyll levels were measured using a spectrophotometer (A646 and A663), and chlorophyll content was determined according to the following formula: total chlorophyll = 20.2 × A645 + 8.02 × A663 (Arnon 1949; Hendry and Grime 1993).

A SPAD chlorophyll meter (SPAD-502, KONICA MINOLTA) was used to estimate the chlorophyll content per unit of leaf area, using a nondestructive method. The SPAD was clipped onto the terminal leaflet of the third leaf.

### Real-time PCR

For quantitative reverse transcription quantitative PCR (RT-qPCR), RNA was extracted from 4 tomato seedlings per biological replicate using the Ribospin Plant kit (GeneAll) from the third leaf (L3) of 21-d-old plants grown under LD (∼170 *µ*mol m^−2^ s^−1^) conditions at 24 °C under white light for 9 d (24WL) and then moved to 24 °C under white light supplemented with far-red light (24FR, R/FR = 0.6) or kept at 24WL. cDNA synthesis was performed using the Maxima first-strand cDNA synthesis kit (Thermo Fisher Scientific) with 1 *µ*g of RNA. RT-qPCR analysis was carried out using StepOnePlus Real-Time PCR Systems (Thermo Fisher), with qPCRBIO SyGreen Blue Mix Hi-ROX (PCR Biosystems). Levels of mRNA were calculated relative to *18S ribosomal RNA* as an internal control (Reuveni et al. 2015). The primers used for the RT-qPCR analysis are listed in Table S1.

### Leaf clearing, sectioning, and microscopy

For the analysis of pavement cells and stomatal index, the terminal leaflet of the third leaf was collected and fixed in 7:1 ethanol:acetic acid overnight to clear the chlorophyll. The cleared leaves were then washed with water and mounted on slides in Hoyer's solution (15 g gum arabic, 10 mL glycerol, 206 g chloral hydrate, 60 mL milliQ water; Liu and Meinke 1998). Leaves were incubated on the slide for a minimum of 24 h before analysis. The abaxial side was inspected using light microscopy (Nikon, ECLIPSE Ni-E), and images of 5 fields of view from 4 leaves per genotype (from 4 different plants) were taken, for a total of 20 images per genotype. To quantify stomatal index, for each field of view, the number of stomata cells was divided by the total number of cells (stomata/[pavement + stomata] × 100).

For the analysis of leaf thickness, the terminal leaflet of the third leaf was fixed in FAA (5% formaldehyde, 50% ethanol, and 5% acetic acid) for 1 h and gradually dehydrated in ethanol (50%, 70%, 90%, 95%, and 100% with eosin), followed by gradual replacement with Histoclear and paraffin embedment (using Paraplast Plus, Leica cat. 39602004). Samples were sectioned using a Leica RM2245 microtome and stained with safranin (Sigma cat. S2255) and Fast Green (Sigma cat. F7258), according to Ruzin (1999). Images were captured under a light microscope (Nikon, ECLIPSE Ni-E) and analyzed using ImageJ (Schindelin et al. 2012). At least 5 leaves were analyzed for each genotype and condition. Images of the shoot apical meristem with young primordia were captured under a light microscope (Zeiss, Axio Imager.A1), and the length of the third leaf was measured using ImageJ.

### Seed germination

Seeds were surface-sterilized with 70% ethanol for 2 min, followed by 3% sodium hypochlorite containing 0.1% Tween 20 for 14 min with rotation, and then rinsed thoroughly with sterile water. Sterilized seeds were imbibed in water for 3 h at room temperature before being sown on Nitsch germination medium (4.4 g L^−1^ Nitsch medium with vitamins; Duchefa, cat. no. N0224, 2% sucrose, and 0.8% agar, pH 5.7). Seeds were incubated at 25 °C under LD white-light conditions, and germination was recorded daily for 8 d. Seeds were considered germinated when the radicle had emerged to ∼2 mm. Experiments were conducted with 6 independent seed batches per genotype, each with 2 replicates. Germination percentage was calculated relative to the total number of seeds germinated by the end of the experiment.

### Accession numbers

Sequence data for genes used in this study can be found in the Sol Genomics Network under the following accession numbers: *SlPIF1a* (*Solyc09g063010*), *SlPIF1b* (*Solyc06g008030*), *SlPIF3* (*Solyc01g102300*), *SlPIF4* (*Solyc07g043580*), *SlPIF7a* (*Solyc03g115540*), *SlPIF7b* (*Solyc06g069600*), *SlPIF8a* (*Solyc01g090790*), and *SlPIF8b* (*Solyc10g018510*), *ATHB2* (*Solyc06g060830*), *CHLM* (*Solyc03g118240*), *POR1* (*Solyc12g013710*), *CAB13* (*Solyc07g063600*), *LHCB3* (*Solyc12g011450*), *HEMA2* (*Solyc04g076870*).

## Supplementary Material

kiag379_Supplementary_Data

## Data Availability

The data underlying this article are available in the article and in its online supplementary material.
