# Supplementary material for: Phytochrome-interacting factors integrate environmental signals to regulate tomato growth and development
Source: Plant Physiol. 2026 Jun 16;201(3):kiag379. doi: 10.1093/plphys/kiag379 (PMC13360281; doi:10.1093/plphys/kiag379)
Supplement: kiag379_Supplementary_Data [file kiag379_supplementary_data.pdf]

Supplementary Figures

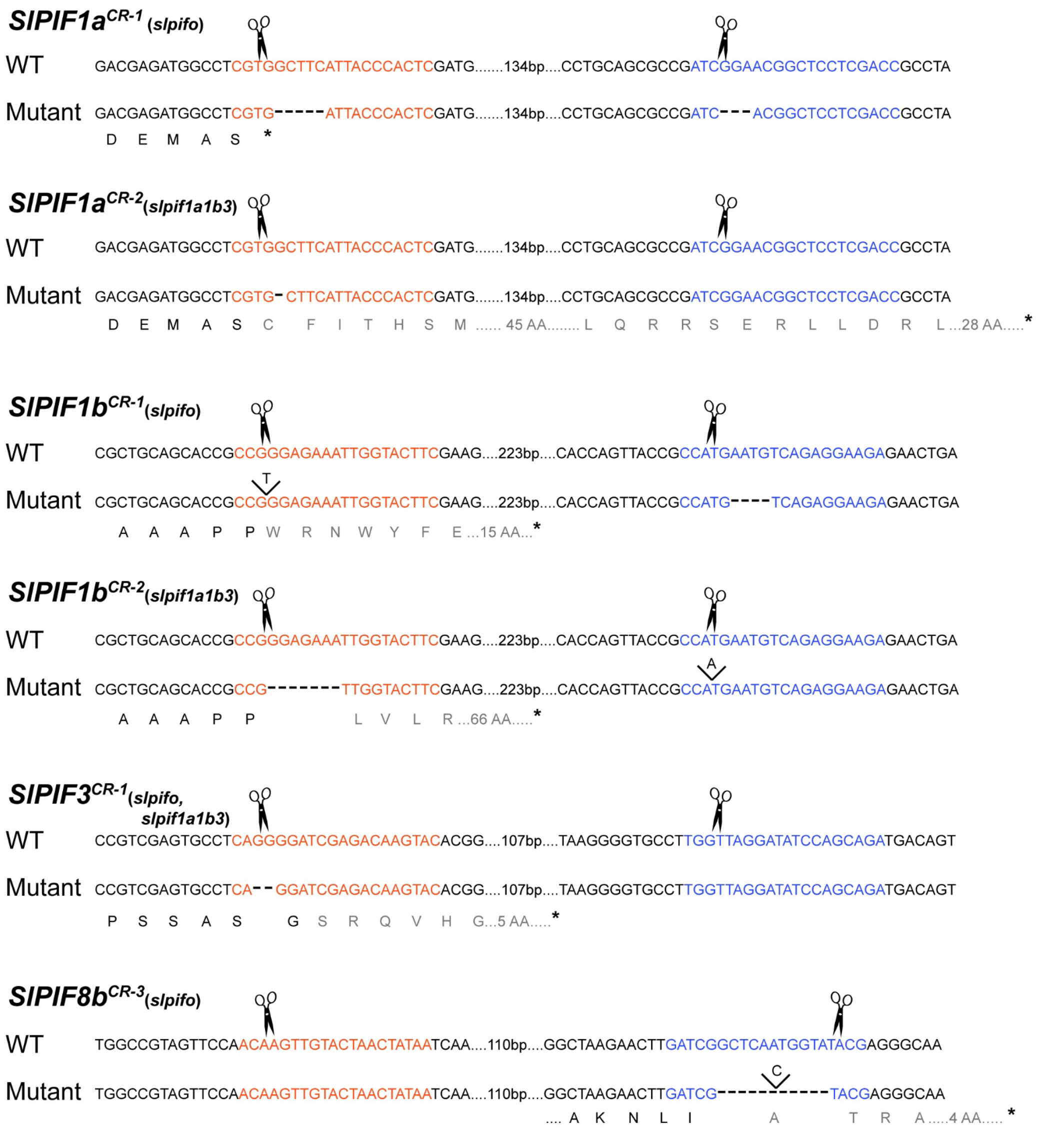

Supplementary Figure S1. CRISPR-edited *SlPIF1a*, *SlPIF1b*, *SlPIF3*, and *SlPIF8b* genes in tomato. Sequences of wild-type (WT, top) and edited *slpif1a*, *slpif1b*, *slpif3*, and *slpif8b* (middle), along with the predicted change in the mutant protein sequence (bottom). All mutations occur in the first third of the gene sequence. The mutant background (*slpif1a1b3* or *slpifo*) containing each CRISPR-generated (CR) allele is indicated in parentheses next to the allele number. The nucleotides in orange and blue represent the binding sites for gRNA1 and gRNA2, respectively. The dashed lines between nucleotides represent deletions (with the number of base pairs deleted). The dotted line represents a gap in the sequence, with the number of nucleotides or amino acids indicated. Scissors indicate the predicted cutting sites in the WT sequence. Black amino acids represent the WT sequence, and gray amino acids represent the expected change in the protein sequence resulting from the CRISPR-mediated change in the gene sequence. An asterisk indicates the newly formed stop codon.

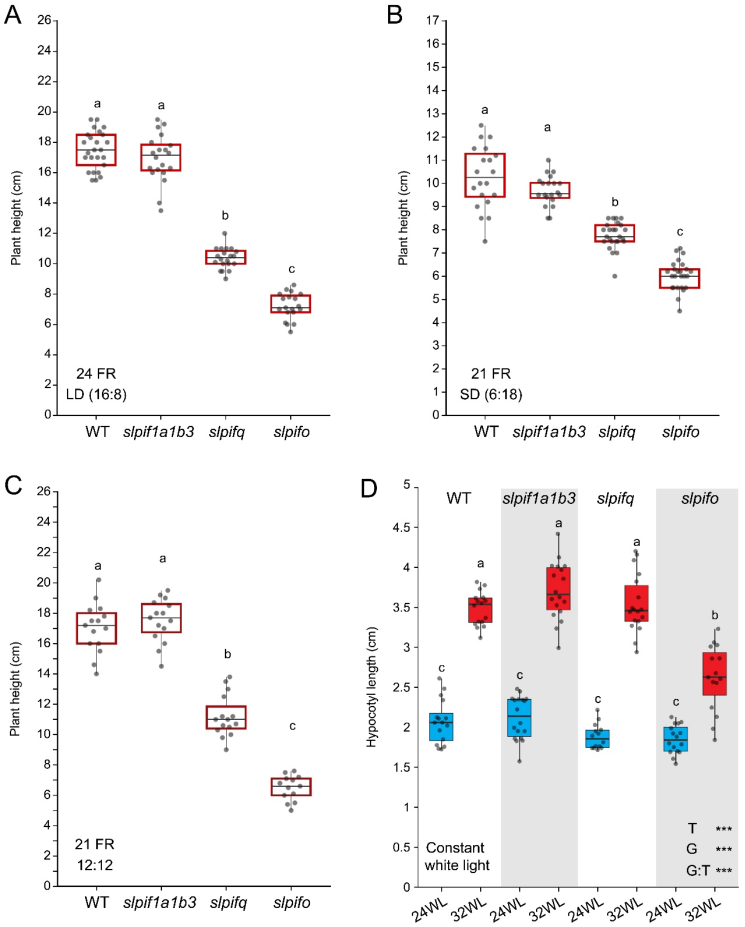

Supplementary Figure S2. The *slpifo* mutant enhances the *slpifq* phenotype in response to low R/FR and affects thermomorphogenesis-induced elongation only in young, media-grown seedlings. **A-C)** Heights of 21-day-old wild-type, *slpif1a1b3*, *slpifq* (*slpif47a7b8a*), and *slpifo* (*slpifq* + *slpif1a1b38b*) mutant plants grown under **(A)** long-day conditions (16 h light / 8 h dark; LD, ~170 μmol m^{-2} s^{-1}), **(B)** short-day conditions (8 h light / 16 h dark; SD, ~200 μmol m^{-2} s^{-1}), or **(C)** day-neutral conditions (12 h light / 12 h dark; 12:12, ~160 μmol m^{-2} s^{-1}) at 24°C **(A)** or 21°C **(B, C)** under white light for 9 days, and then moved to the same light conditions plus supplemented of far-red light (R/FR = 0.6). n > 13 plants per sample. **D)** Hypocotyl length of 7-day-old wild-type and *slpif*-mutant seedlings grown on plates containing Nitsch medium. Seeds were germinated under LD white light (~200 μmol m^{-2} s^{-1}) at 24°C for 3 days and then transferred to either 32°C (32WL) or 24°C (24WL) constant white light conditions. n > 14 seedlings per sample. Boxes indicate the first and third quartiles, whiskers indicate the minimum and maximum values, black lines within the boxes indicate the median values, and gray dots indicate the individual data points. Different letters denote statistical differences ($p < 0.05$) among samples, as assessed by one-way (**A-C**) or two-way (**D**) ANOVA and Tukey's HSD. T, temperature; G, genotype; G:T, interaction between genotype and temperature. ***$p < 0.001$.

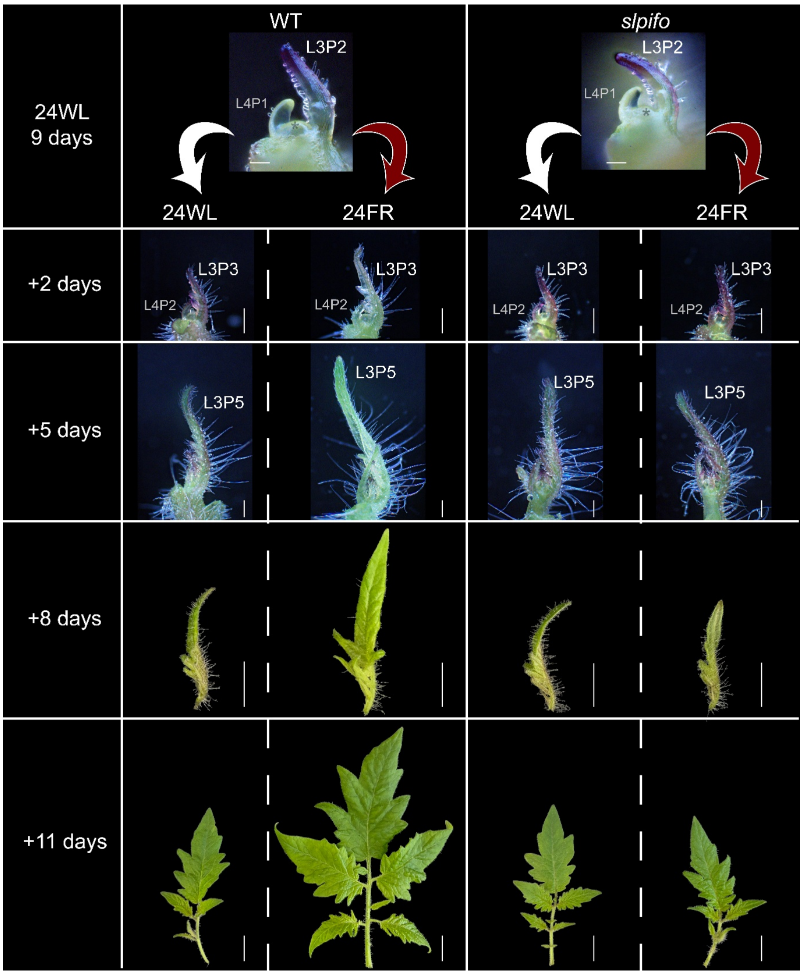

Supplementary Figure S3. Low R/FR conditions modulate third-leaf growth and development through SlPIF-dependent mechanisms. Representative images of the third leaf from wild-type and *slpifo* mutant plants at the indicated time points. The plants were grown under long-day (~170 µmol m^{-2} s^{-1}) conditions at 24°C under white light for 9 days (24WL), and then moved to 24°C under white light supplemented with far-red light (24FR, R/FR = 0.6) or kept at 24WL. Developmental stages marked by plastochrons (P) are shown in images taken before the plants were moved between conditions (24WL, 9 days) and at 2 and 5 days after they were moved (+2 days, +5 days) to the indicated conditions. Plastochrons represent intervals between successive leaf primordia. P1 denotes the youngest visible leaf primordium; it becomes P2 when the next primordium is initiated, and so forth. L3 and L4, the third and fourth true leaves, respectively. *, shoot apical meristem. Scale bars: 100 µm for 24WL 9 days, 500 µm for +2 days and + 5 days, 5 mm for +8 days, and 1 cm for +11 days. The images from days +8 and +11 were digitally extracted for comparison.

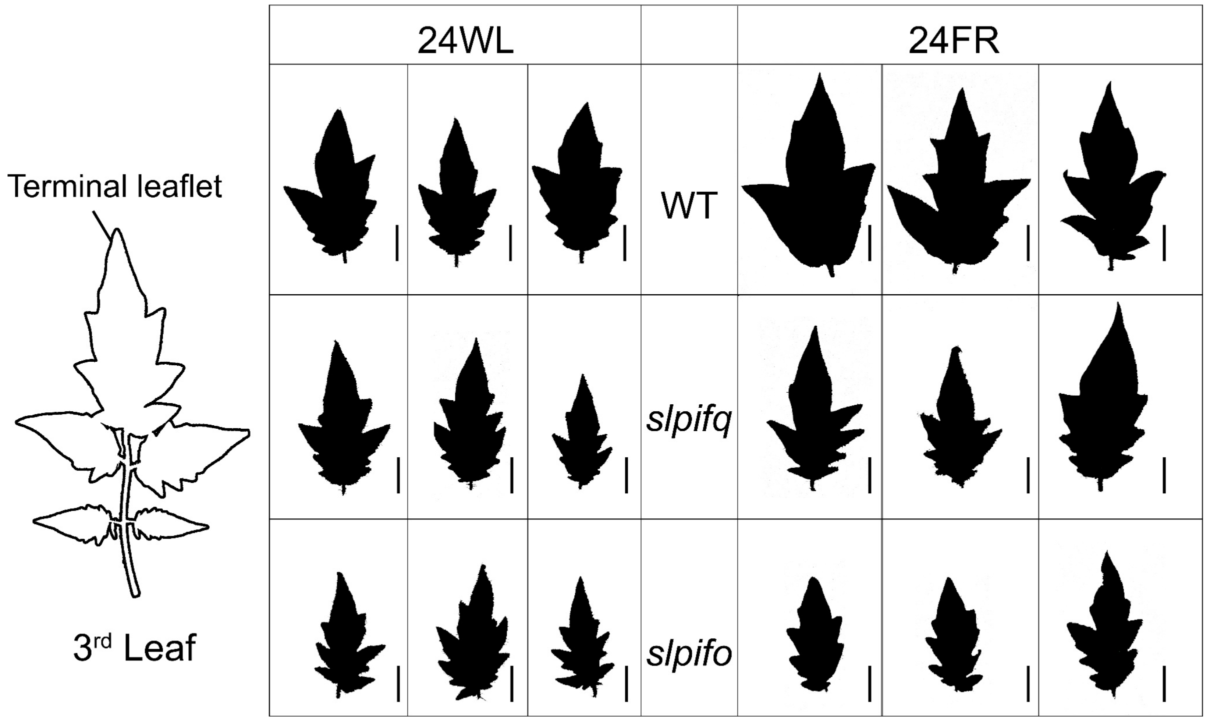

Supplementary Figure S4. Low R/FR conditions alter terminal leaflet growth in an SlPIF-dependent manner. Representative terminal leaflets of the third leaf from 21-day-old wild-type, *slpifq*, or *slpifo* mutant plants grown under long-day (~170 μmol m^{-2} s^{-1}) conditions at 24°C under white light for 9 days (24WL), and then moved to 24°C under white light supplemented with far-red light (24FR, R/FR = 0.6) or kept at 24WL. Scale bars: 1 cm. Images were digitally extracted for comparison. See Fig. 2A for quantification of leaflet area.

.

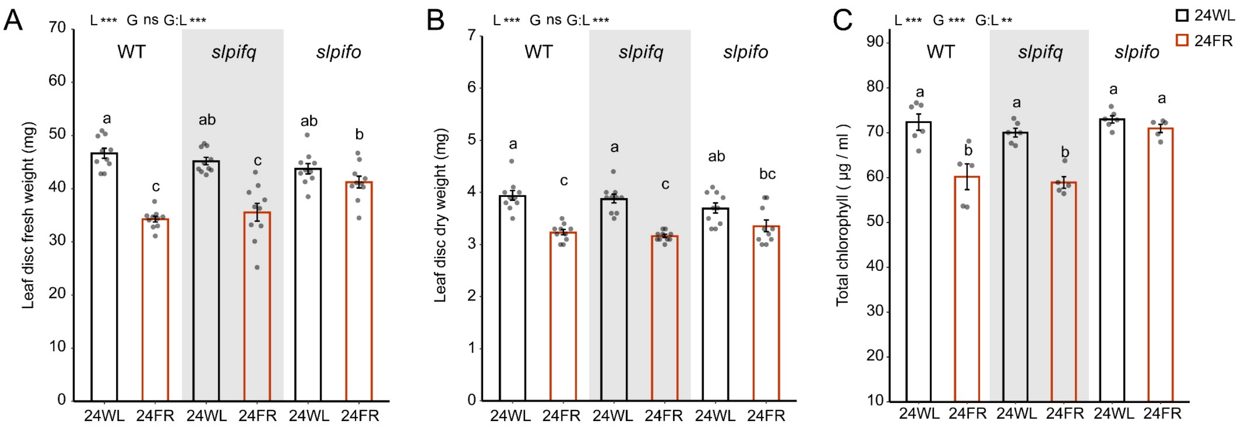

Supplementary Figure S5. Low R/FR light conditions affect both leaf thickness and chlorophyll depletion in an SlPIF-dependent manner. **A)** Leaf disc fresh weight, **(B)** dry weight, and **(C)** total chlorophyll content per milliliter of extract, of the indicated genotypes and conditions. Measurements were performed on terminal leaflets of the third leaf (L3) from 21-day-old tomato plants grown under long-day (~170 µmol m−2 s−1) conditions at 24°C under white light for 9 days (24WL), and then moved to 24°C under white light supplemented with far-red light (24FR, R/FR = 0.6, red bars) or kept at 24WL (black bars). The average values ± *SE* are presented. *n* > 5. Each biological replicate consisted of three leaf discs collected from three different plants. Different letters denote statistical differences (*p* < 0.05) among samples, as assessed by two-way ANOVA and Tukey's HSD. L, light; G, genotype; G:L, interaction between genotype and light. ***p* < 0.01; ****p* < 0.001; ns, not significant. Gray dots indicate the individual data points.

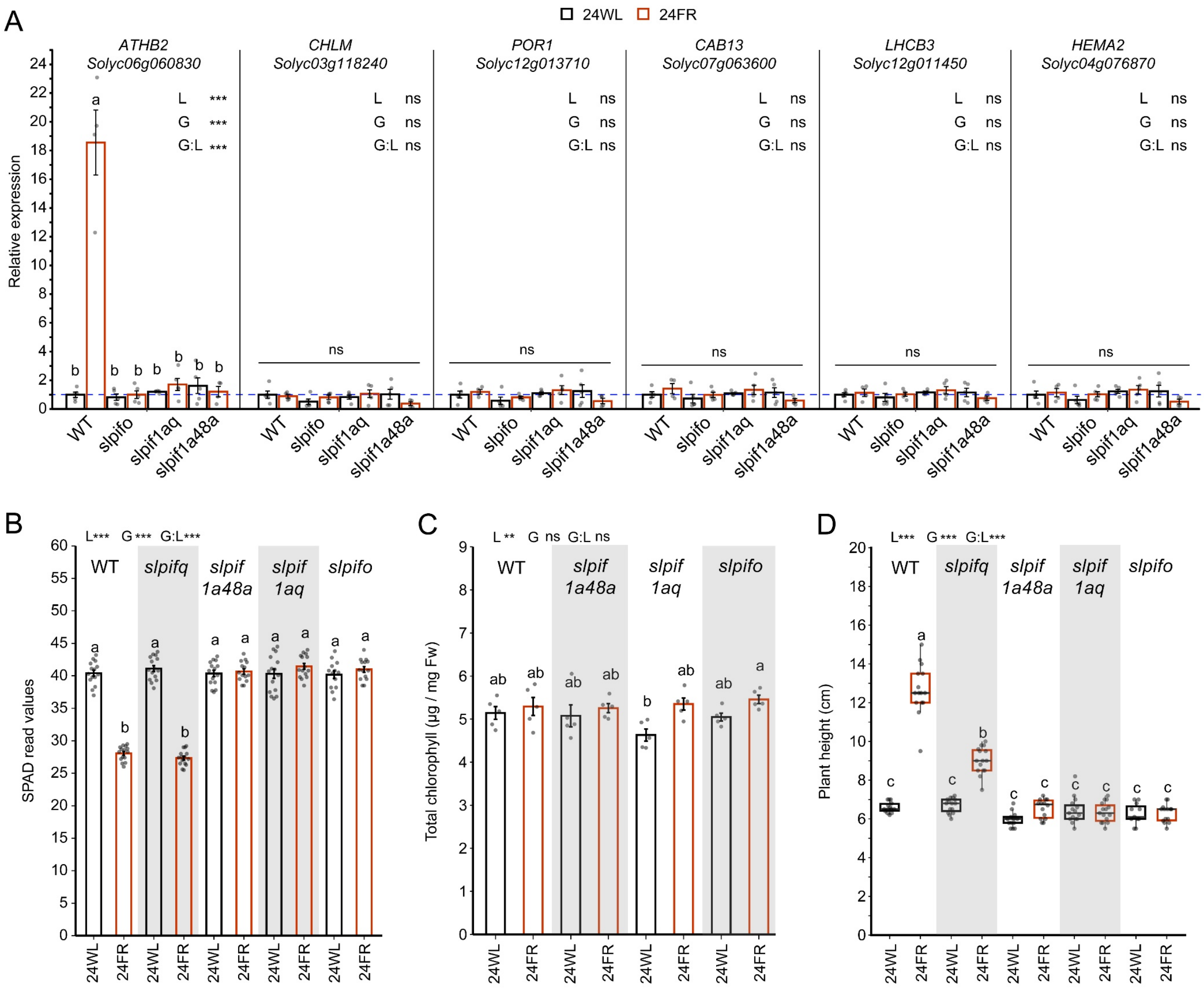

Supplementary Figure S6. In tomato, low R/FR promotes shade avoidance responses in an SlPIF1a48a-dependent manner. **A)** Relative expression of the indicated genes in wild-type and *slpif* mutants in the third leaf (L3) of 21-day-old plants grown under 24WL (black bars) or 24FR (red bars), as described in Supplementary Fig. S5. Gene expression was assayed using RT-qPCR and normalized for each gene separately relative to the *18S ribosomal RNA* and to the expression level in WT at 24WL. The average values of at least 4 biological replicates per condition ± SE are shown. The blue dashed line indicates the normalized level of the WT at 24WL. **B)** Chlorophyll content per unit of leaf area measured using SPAD, and **(C)** total chlorophyll content per unit of fresh weight. Measurements were performed on terminal leaflets of the third leaf (L3) from tomato plants exposed to either 24WL (black bars) or 24FR (red bars) for 12 days, as described in Supplementary Fig. S5. The average values ± *SE* are presented. *n* = 5. In **C**, each biological replicate consisted of three leaf discs collected from three different plants. **D)** Heights of 21-day-old wild-type and *slpif*-mutant plants grown under 24WL (black boxes) or 24FR (red boxes) as described in Supplementary Fig. S5. *n* > 11 plants per sample. Boxes indicate the first and third quartiles,

whiskers indicate the minimum and maximum values, black lines within the boxes indicate the median values, and gray dots indicate the individual data points.

In **A-D**, different letters denote statistical differences (p < 0.05) among samples, as assessed by two-way ANOVA and Tukey's HSD. L, light; G, genotype; G:L, interaction between genotype and light. **p < 0.01; ***p < 0.001; ns, not significant. Gray dots indicate the individual data points.

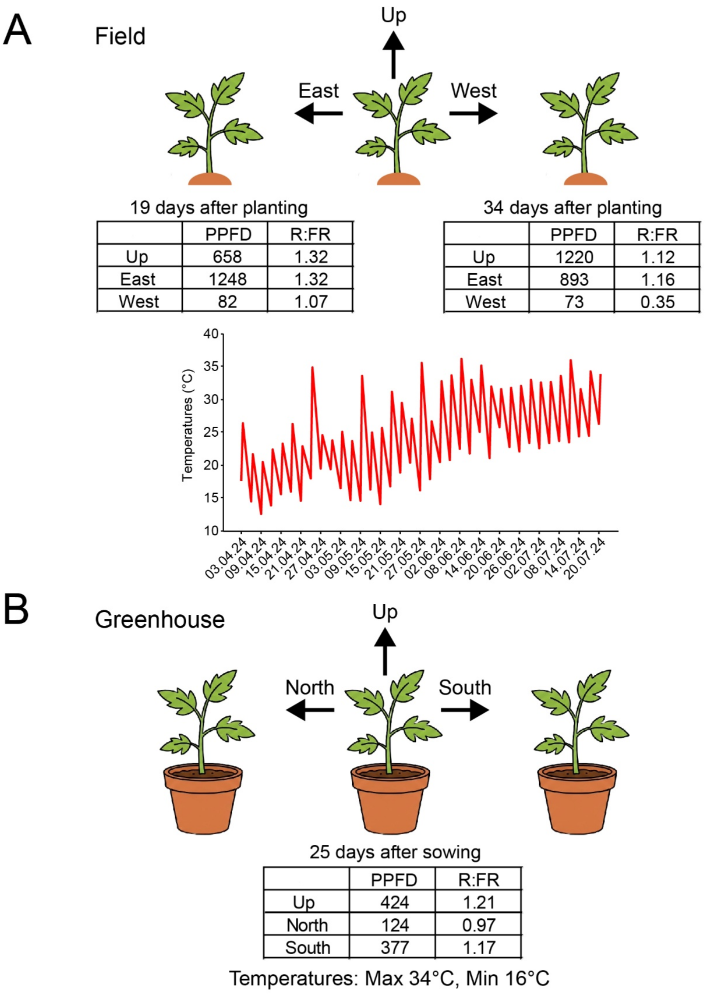

Supplementary Figure S7. Growth conditions in the greenhouse and open-field experiments. **A-B**) Changes in light quality and quantity in close proximity to the plants, as well as the temperature range, are shown for the greenhouse (**A**) and open-field (**B**) experiments. PPFD (Photosynthetic Photon Flux Density in μmol m^{-2} s^{-1}) and R:FR values were measured, with a LI-COR LI-180 spectrometer. Arrows indicate the directions of the light measurements relative to the tested plant and the orientation of the growing row. In **B**, the maximum and minimum temperatures throughout the growing season are shown.

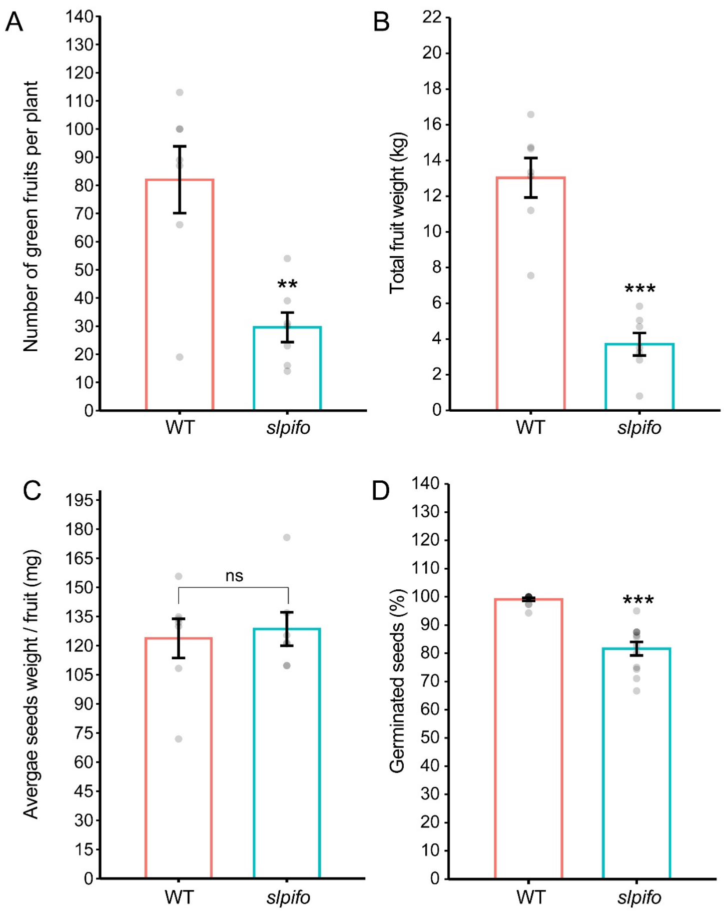

Supplementary Figure S8. Tomato plants lacking all active SlPIFs exhibit reduced productivity. **A)** Number of green fruits per plant, **(B)** total fruit weight, and **(C)** average seed weight per fruit from wild-type and *slpifo* mutant plants grown under open-field conditions. The average values of 7 biological replicates per genotype ± *SE* are presented. **D)** Percentage of wild-type and *slpifo* seeds that germinated after 8 days (see Fig. 5I for germination kinetics). The average values of at 12 biological replicates per genotype ± SE are shown. In **D**, 6 independent seed batches were used for each genotype. In **A-D**, $^{**}p < 0.01$; $^{***}p < 0.001$, ns, not significant, according to Student's *t*-test. Gray dots indicate the individual data points.

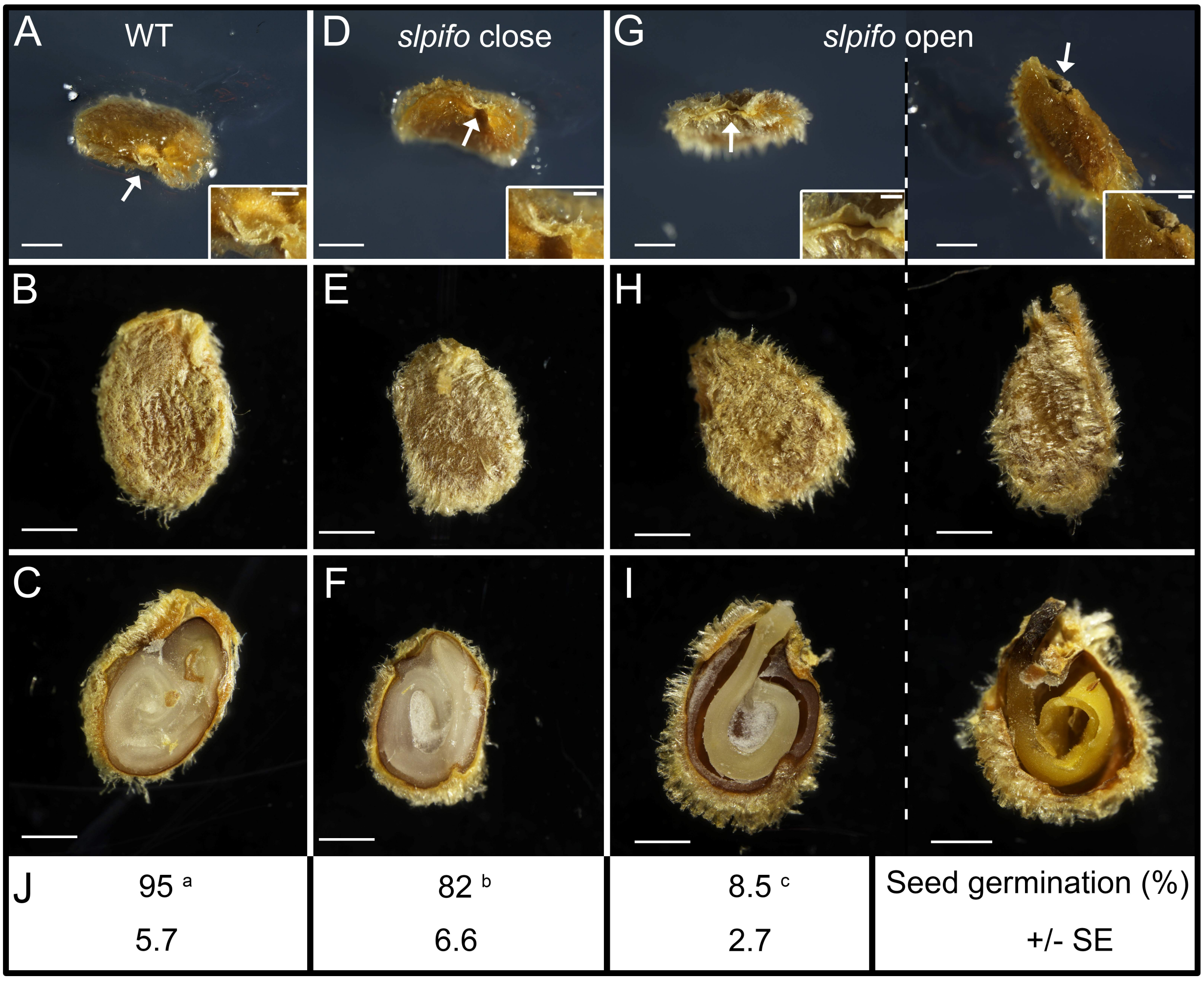

J				
	95 [a]	82 [b]	8.5 [c]	Seed germination (%)
	5.7	6.6	2.7	+/- SE

Supplementary Figure S9. The *slpifo* seeds with an open cap display reduced germination. **A-I)** Representative images of WT **(A-C)**, *slpifo* seeds with a closed cap **(D-F)**, and *slpifo* seeds with an open cap **(G-I)**. **B, E, H)** Representative external views of dry seeds. **C, F, I)** Representative longitudinal sections of peeled-open seeds, showing embryo and seed internal structures. In **A**, **D**, and **G**, arrows indicate the seed cap, which is shown at higher magnification in the inset. **J)** Germination percentage of WT, *slpifo* seeds with a closed cap, and *slpifo* seeds with an open cap that germinated after 8 days. The average values ± *SE* are presented. $n = 4$. Different letters denote statistical differences ($p < 0.05$), as assessed by one-way ANOVA and Tukey's HSD. Scale bars: 1 mm for A-I and 0.25 mm for the inset.

A

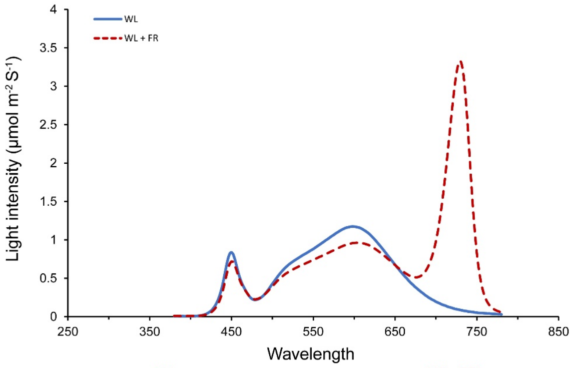

WL

B	R	FR	R:FR	PAR
30	69	7	10.48	187

WL+FR

B	R	FR	R:FR	PAR
27	74	118	0.63	174

B

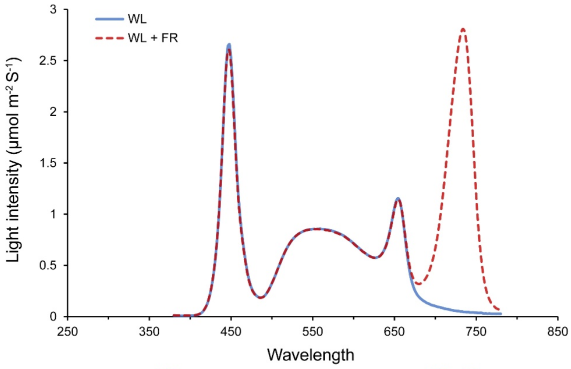

WL

B	R	FR	R:FR	PAR
70	57	4	14.13	202

WL+FR

B	R	FR	R:FR	PAR
69	64	106	0.60	208

C

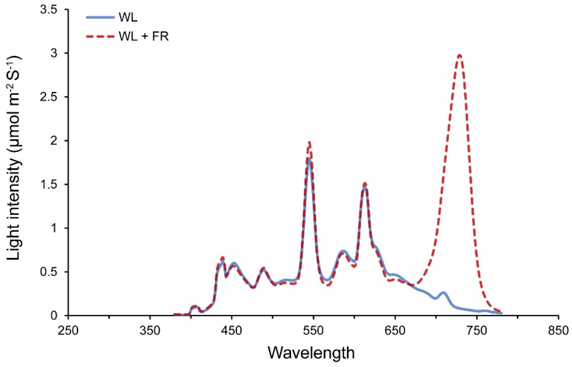

WL

B	R	FR	R:FR	PAR
36	57	8	7.20	159

WL+FR

B	R	FR	R:FR	PAR
36	62	105	0.60	163

D

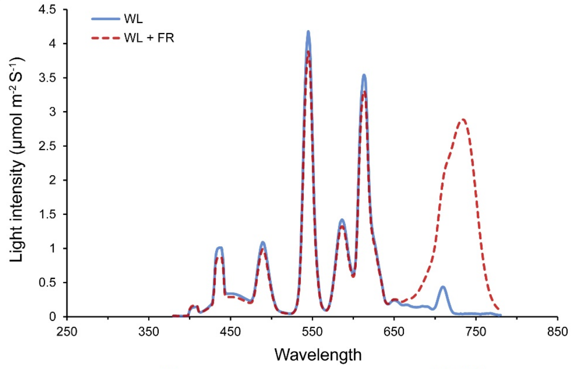

WL

B	R	FR	R:FR	PAR
43	74	8	8.75	205

WL+FR

B	R	FR	R:FR	PAR
38	83	135	0.62	204

Supplementary Figure S10. Spectral distributions of the light used in the white-light and low-R/FR conditions. **A-D**) Spectral distributions of white light (WL, blue line) and white light supplemented with far-red light (WL + FR, red dashed line), measured with a LI-COR LI-180 spectrometer and present in μmol m^{-2} s^{-1}. The spectrum shown in **A** was used for the long-day (LD, 16 h light / 8 h dark) experiments conducted at 24°C (Figs. 2-4, and Supplementary Figs. S2A, S3-6). The spectrum shown in **B** was used for the LD experiments conducted at 21°C, 26°C, 30°C and 34°C (Fig. 1A, B, E, F), and the white light spectrum was used for the warm temperature under constant white light experiment (Supplementary Fig. S2D). The spectrum shown in **C** was used for the 12 h light / 12 h dark experiments conducted at 21°C and 30°C (Fig. 1D and Supplementary Fig. S2C). The spectrum shown in **D** was used for the short-day (SD, 8 h light / 16 h dark) experiments conducted at 21°C and 30°C (Fig. 1C and Supplementary Fig. S2B). B, Blue (400-500 nm); R, red (600-700 nm); FR, far-red (700-800 nm); PAR (photosynthetically active radiation, 400–700 nm).

Supplementary Table S1. Primers used in this work

Primer name	Sequence 5' >>> 3'	Purpose	Use
SlPIF1a_gRNA_1	TGTGGTCTCAATT**GAGTGGGTAATGAAGCCACG**GTTTTAGAGCTAGAAATAGCAAG	Cloning SlPIF1a gRNAs with universal primer	Transform into tomato
SlPIF1a_gRNA_2	TGTGGTCTCAATT**GGTCGAGGAGCCGTTCCGAT**GTTTTAGAGCTAGAAATAGCAAG		
SlPIF1b_gRNA_1	TGTGGTCTCAATT**GAAGTACCAATTTCTCCCGG**GTTTTAGAGCTAGAAATAGCAAG	Cloning SlPIF1b gRNAs with universal primer	
SlPIF1b_gRNA_2	TGTGGTCTCAATT**TCTTCCTCTGACATTCATGG**GTTTTAGAGCTAGAAATAGCAAG		
SlPIF3_gRNA_1	TGTGGTCTCAATT**GTACTTGTCTCGATCCCCTG**GTTTTAGAGCTAGAAATAGCAAG	Cloning SlPIF3 gRNAs with universal primer	
SlPIF3_gRNA_2	TGTGGTCTCAATT**TCTGCTGGATATCCTAACCA**GTTTTAGAGCTAGAAATAGCAAG		
SlPIF8b_gRNA_1	TGTGGTCTCAATT**GATCGGCTCAATGGTATACG**GTTTTAGAGCTAGAAATAGCAAG	Cloning SlPIF8b gRNAs with universal primer	
SlPIF8b_gRNA_2	TGTGGTCTCAATT**TTATAGTTAGTACAACTTG**TGTTTTAGAGCTAGAAATAGCAAG		
gRNA_universal_primer	TGTGGTCTCAAGCGTAATGCCAACTTTGTAC	PCR with gRNA primers	
SlPIF1a det- F	GATGCGGTTATTCCCTCTGA	Genotype *SlPIF1a*	Genotyping
SlPIF1a det- R	GGCACTGGCGCTTACACTAT		
SlPIF1b det- F	TGATGATTCCTCCTTCGATGA	Genotype *SlPIF1b*	
SlPIF1b det- R	CTGTGGCGGTCCTGCTCT		
SlPIF3 det- F	TGCAGTCCTGAGAATGACTTG	Genotype *SlPIF3*	
SlPIF3 det- R	TGAATCCCCAATCATCTTGTC		
SlPIF8b det- F	GTGACGAGGGTGGTGATGAT	Genotype *SlPIF8b*	
SlPIF8b det- R	TGTGCTGCATGGGAATCATA		
CAS9 F	CGCTAATCTTGCAGGTAGCC	Genotype for CAS9-free plants	
CAS9 R	TGCCAGCTCGTTACCTTTCT		
ATHB2_RT_F	TTGGCTTTGGCAAAAAGACT	*Solyc06g060830*	RT-qPCR
ATHB2_RT_R	TTGCTTCAATTTTGTCCTAGCTC		
CHLM_RT_F	TGCTATTCTATCGCTCGCCG	*Solyc03g118240*	
CHLM_RT_R	CACCACCTCCTTATCACCGC		
POR1_RT_F	GACCTTGCATCGCTTGACAG	*Solyc12g013710*	
POR1_RT_R	GAAGGCTCTTTCGCGGTAGG		
CAB13_RT_F	ATGCTTGGAGCTTTTGGTTGC	Solyc07g063600	
CAB13_RT_R	TTGCCCAAATAGTCCAGCCC		
LHCB3_RT_F	CCAAGTGGTGCTTATGGGACT	Solyc12g011450	
LHCB3_RT_R	ATTGGCCACCGGGGTATAAG		
HEMA2_RT_F	GCAGGCGCAGTTTCTGTAAG	Solyc04g076870	
HEMA2_RT_R	CCTAGCAGTAGTGTGGCAGG		
18S_RT_F	GCGACGCATCATTCAAATTTC	18S ribosomal RNA	
18S_RT_R	TCCGGAATCGAACCCTAATTC		
